# Collaboration–competition dilemma in flattening the COVID‐19 curve

**DOI:** 10.1111/poms.13709

**Published:** 2022-04-14

**Authors:** Kim E. Van Oorschot, Luk N. Van Wassenhove, Marianne Jahre

**Affiliations:** ^1^ Department of Accounting and Operations Management BI Norwegian Business School Oslo Norway; ^2^ Technology and Operations Management, INSEAD Fontainebleau France

**Keywords:** collaboration, COVID‐19, diagnostic testing, supply chain management, system dynamics

## Abstract

Testing for COVID‐19 is a key intervention that supports tracking and isolation to prevent further infections. However, diagnostic tests are a scarce and finite resource, so abundance in one country can quickly lead to shortages in others, creating a competitive landscape. Countries experience peaks in infections at different times, meaning that the need for diagnostic tests also peaks at different moments. This phase lag implies opportunities for a more collaborative approach, although countries might also worry about the risks of future shortages if they help others by reallocating their excess inventory of diagnostic tests. This article features a simulation model that connects three subsystems: COVID‐19 transmission, the diagnostic test supply chain, and public policy interventions aimed at flattening the infection curve. This integrated system approach clarifies that, for public policies, there is a time to be risk‐averse and a time for risk‐taking, reflecting the different phases of the pandemic (contagion vs. recovery) and the dominant dynamic behavior that occurs in these phases (reinforcing vs. balancing). In the contagion phase, policymakers cannot afford to reject extra diagnostic tests and should take what they can get, in line with a competitive mindset. In the recovery phase, policymakers can afford to give away excess inventory to other countries in need (one‐sided collaboration). When a country switches between taking and giving, in a form of two‐sided collaboration, it can flatten the curve, not only for itself but also for others.

## 1 INTRODUCTION

The COVID‐19 pandemic has stressed hospitals and intensive care units (ICUs), disrupted supply chains for most goods and services, eliminated jobs, and threatened people's livelihoods (OECD, 2020). Because interventions to prevent the worldwide outbreak (such as strict containment) came too late, in the spring of 2020, policymakers shifted their focus to minimizing the virus's impact by flattening the infection curve (Bechteva et al., 2020) through such means as social distancing, lockdowns, and testing. Most countries managed to flatten the curve in the first half of 2020, implying that the combination of efforts was helping. However, there was no time to perform thorough analyses of each intervention, so most countries tried them all, without any evidence of which (combination of) interventions worked best (Gibney, 2020). This challenge has persisted into the pandemic's subsequent waves (Rogers, 2020). Global society has been unprepared for the effects of this interconnected dynamic system, in which mistakes are expensive or deadly. The exponential character of the infection and relatively long lead times between policy decisions and outcomes (such as obtaining good health information), together with unexpected supply chain delays, create massive difficulties. Such challenges cannot be addressed without understanding the interconnections of different subsystems (policymaking, health management, and supply chain management), which is the motivation for the current research.

We propose a method for studying the key interconnections and addressing their dynamic complexity. With a few recent exceptions (Büyüktahtakin et al., 2018), the production, operation, and supply chain management literature has not accounted sufficiently for such dynamics (Paul & Venkateswaran, 2020). The epidemiological literature has investigated the effects of individual and combined interventions (for a review, see Dasaklis et al., 2012), such as the outcomes of setting up treatment centers and allocating household protective kits during the 2014 Ebola outbreak (Lewnard et al., 2014; Merler et al., 2015). However, such studies typically assume zero lead times and automatic replenishment, without accounting for supply chain activities and possible delays (Paul & Venkateswaran, 2018). They also often focus on single systems (Chang et al., 2017), unlike classic system dynamic models that include interactions among multiple subsystems. A notable exception is Duintjer Tebbens et al. (2010) and other works by him and his colleagues (see Duintjer Tebbens & Thompson, 2018).

Such considerations need to be combined to analyze the availability of COVID‐19 diagnostic tests because they represent a scarce, finite global resource (Seidu et al., 2020). A positive test initiates interventions such as the isolation of infected people and contact tracing to identify other potentially infected persons to prevent further spreading. Therefore, having sufficient diagnostic tests has become a critical goal during the pandemic. In seeking sufficiency, however, some countries attained an outright abundance (Kavanagh et al., 2020), which contributed to shortages in other countries. A similar process happened more recently with COVID‐19 vaccines: “As some richer countries hoard vaccines, they make a mockery, frankly, of vaccine equity” (WHO, 2021). In the beginning of the pandemic, access to diagnostic tests became a competition, intensified by capacity constraints due to insufficient sources of supply, which created dire shortages, long lead times, and nonequitable fights for supplies. Furthermore, countries suffered the strongest pandemic effects at different times, so their need for diagnostic tests also peaked variously. Although a lack of personnel or machines for laboratory analyses became an issue in some cases (Behnam et al., 2020), test shortages clearly contributed to delays. Finally, multiple waves already have emerged, such that countries continue to experience demand swings at different times. Such phase lags suggest the promise of more collaborative approaches at a regional or even global level.

So what happens if countries collaborate on diagnostic test supply? Formally, the research question that drives this study is: *What are the risks to countries that help one another by reallocating their excess inventory of diagnostic tests?* We propose a simulation model to answer this question, in which three subsystems interconnect: the transmission of COVID‐19, the diagnostic test supply chain, and public policy interventions aimed at flattening the curve. Our focus is not on *how* countries should collaborate but rather on establishing that it would make sense to do so. Hence, our study does not go into issues related to challenges and drivers for *implementing* collaborative approaches.

To validate the proposed model, we use real open‐access data, that is, actual infection data from Norway obtained from publications by the Norwegian Institute of Public Health. We also checked newspaper articles and publications by health organizations to verify statements about diagnostic test availability and create simulated inventory gaps. We simulate a competitive policy (take what you need), a one‐sided collaborative policy (give when you can), and a two‐sided collaborative policy (take what you need, give when you can). We then compared the results of the simulations with a base case of an isolation policy (neither take nor give), which is what truly happened in Norway and led to surpluses of diagnostic tests when they were not needed but also to shortages when they were needed.

We established that two‐sided collaboration is best because it flattens the curve while offering acceptable levels of risk (not self‐sacrificing) and establishing equity (donating to countries in need). As expected, access to more diagnostic tests helps, but beyond this confirmation, we determined that donating excess supply does not have a negative impact on the curve in a particular country. In this way, we provide a clearer understanding of the whole system and demonstrate the need to combine policymaking, health, and supply chain expertise to deal with a pandemic situation.

Furthermore, this study answers calls for more real‐world case studies (Paul & Venkateswaran, 2020) that integrate disease‐specific supply chains with epidemic models. Our findings can also inform research on horizontal cooperation in a disaster relief context, which remains underdeveloped (Toyasaki et al., 2017). Finally, by studying the sequential process of taking and giving, we offer a clear illustration of the interplay of competition and cooperation, or “sequential coopetition.” This insight represents a response to Hoffman et al.’s (2018) call for more research into the *temporal* dimension of such relations as partners shift between roles as competitors or collaborators.

## 2 LITERATURE REVIEW

### 2.1 Combining epidemics and supply chain management

The production, operations, and supply chain management (POSCM) literature generally does not account for complex, dynamic situations, such as a pandemic, in which a supply chain bottleneck (such as diagnostic tests) can accelerate the propagation of a threat (such as disease) in a nonlinear fashion. Demand both affects and is affected by the supply chain. In recent studies that incorporate interactions of disease and operations, De Vries et al. (2016) noted that the planning of mobile screening operations can affect and is affected by disease eradication. Büyüktahtakın et al. (2018) introduced a model that simultaneously captures the spatial spread of an epidemic and logistics that can be applied to other infectious diseases; they provide tangible policy recommendations for controlling infectious disease outbreaks over large spatial and temporal scales. In an integrated model, Paul and Venkateswaran (2018) further showed that epidemic data alone do not suffice to estimate actual disease infectivity, such that corresponding medicine supply chain information (delays, number of echelons, ordering policies) is also necessary. Kochan et al. (2018) used system dynamics to develop two conceptual causal loop diagrams, one representing traditional and the other cloud‐based information sharing in a hospital supply chain. They found that the latter improves visibility and reduces the impact of shortages. Paul and Venkateswaran (2020) compared the robustness of three supply chain ordering policies and determined that the ordering policy parameters, lead time, and safety stock coverage are critical, such that aggressive ordering policies and the removal of intermediate echelons significantly reduces the impact of epidemics. Finally, Araz et al.’s (2020) conceptual framework aims to address COVID‐19 testing challenges by combining disease dynamics and epidemiology with logistics. All these studies called for more research.

In response, we focus specifically on the interaction of disease and supply chain dynamics and treat the demand and supply of diagnostic tests as endogenous. For example, a sudden increase in demand can lead to a temporary shortage of such supply. This shortage increases the infection rate and thus increases the future demand for diagnostic tests. Our approach also includes public policy as a third dynamic factor. Most policymakers do not just wait for diagnostic tests to arrive or ignore the outbreak; they seek other interventions, such as (partial) lockdowns, and those interventions also influence both demand and supply for diagnostic tests. Responding to the call issued by Araz et al. (2020), we propose a model that can capture such complex, dynamic interactions of supply chain management, the pandemic, and public policy interventions.

Outside of the POSCM literature, relevant papers accounting for system complexity provide evidence‐based decision support for the eradication of vaccine‐preventable diseases. Duintjer Tebbens and Thompson (2018) discussed the use of integrated models for epidemiological risk management, combining transmission modeling with health economics and stockpile optimization. Duintjer Tebbens et al. (2010) combined a vaccine supply chain model with a polio transmission model to demonstrate how lack of supply increases demand and how issues such as capacity constraints, delays, risks of stockpiling, and location impact the supply and thus the demand. Similarly, Thompson and Duintjer Tebbens (2016) and Duintjer Tebbens and Thompson (2019) combined supply and transmission models to demonstrate investment trade‐offs and discussed how a lack of coordination at the regional and global levels may lead to competition between proactive (vaccination regardless of incidence) and reactive (allocation depending on incidence and urgency) efforts for the supply of vaccines in an outbreak response. However, none of these studies has explicitly integrated supply chain and transmission models with public policy models. For example, Thompson et al. (2021, p. 1) suggested two key roles for health systems to control transmission, that is, to “flatten the curve”: (1) treating the symptoms of infection to improve health outcomes and (2) preventing transmission by isolating highly infectious individuals. The present study is concerned with the latter.

### 2.2 Collaboration and competition in supply chains

POSCM research focuses less on horizontal than on vertical supply chain collaboration (Pomponi et al., 2017). Defined as a collaboration by actors at the same supply chain level, horizontal collaboration might take the form of group purchasing, shared inventory locations, or joint transportation. It typically requires judicious switching between collaboration and competition, commonly termed coopetition, and the combination of different interaction logics: conflicting interests and hostility in a competitive setting versus trust and friendliness in pursuing a common, collaborative goal (Stadtler & Van Wassenhove, 2016). This interplay features a strong *temporal* dimension, such that actors move between competition and cooperation, which is termed sequential coopetition (Hoffman et al., 2018).

Horizontal collaboration to share emergency stocks is a well‐established disaster relief tactic (Van Wassenhove, 2006). Toyasaki et al. (2017) considered the incentives that humanitarian organizations have to cooperate, such as prepositioning relief items in a common warehouse, allowing for stock transshipments, or loaning and borrowing to pool risks and reduce costs. In a similar vein, Balcik et al. (2019) proposed a collaborative prepositioning network for Caribbean countries to achieve risk‐pooling benefits because different countries are affected each hurricane season. Historical data indicate that sufficient diversity exists to achieve risk‐pooling benefits and that resource sharing might help countries cope with the immediate consequences of disasters. Building on this study, Rodriguez‐Pereira et al. (2021) analyzed solidarity in the region in terms of cost allocation considering disaster risk and economic standing parameters.

However, if policymakers sense they are competing for scarce resources, like COVID‐19 diagnostic tests, it is unlikely that collaboration will be possible or appealing. On the contrary, the instinctive reaction for countries hit by high infection rates was to close borders and focus on their local issues. Mehrotra et al. (2020) modeled how US states shared ventilator stocks during COVID‐19 and explained that “Each participating state is risk averse to sharing their excess inventory at any given time” (p. 317). During crises, policymakers tend to be risk‐averse, favoring a competitive approach over a collaborative one.

With the exception of a few studies within disaster relief, evidence‐based research with real data on horizontal cooperation is scarce (Toyasaki et al., 2017). Such research needs to answer two questions: (1) What are the benefits and risks of such collaboration, and (2) if the benefits outweigh the risks, what does it take to establish such a collaboration? Our paper deals with the first question, not the second.

## 3 APPROACH

### 3.1 Introduction

Grounded in the system dynamics approach, we develop a simulation model to capture and connect three subsystems: COVID‐19 transmission, diagnostic test supply chain, and public policy interventions. The relationships in the model are generic, but we calibrate this model with Norwegian data on infections, hospitalizations, and the timing of policy interventions (such as start and end days of the partial lockdowns). With the calibrated model, we also perform “what‐if” simulations to analyze the impact of having the right number of diagnostic tests at the right point in time on the infection curve. The different scenarios mimic the impacts of horizontal collaboration in the form of exchanges of diagnostic tests among countries. Both the simulation model and the full model description, including the calibration process, are available as Supporting Information (Appendices 1–4). In the following sections, we present a high‐level overview of the simulation model and summarize the calibration process.

### 3.2 Model description

**FIGURE 1:**
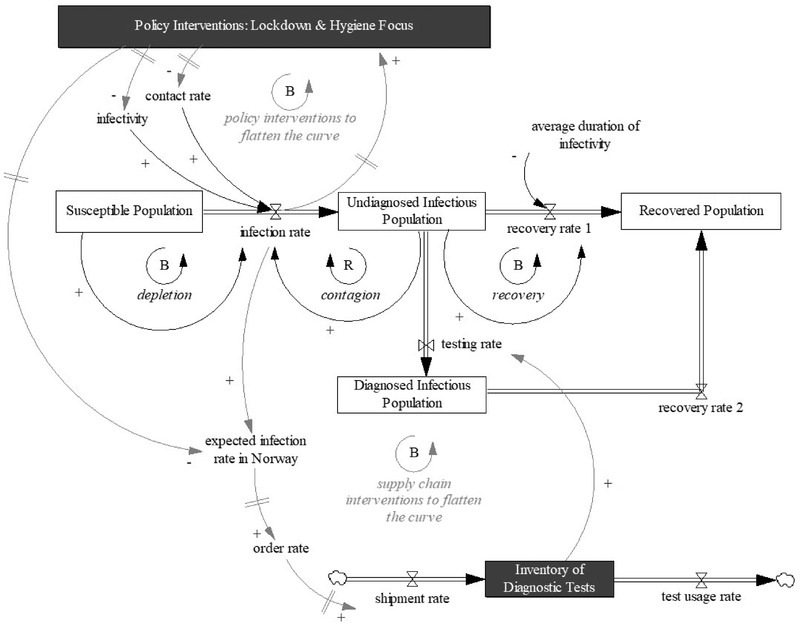
High‐level overview of the model

Figure 1 contains a high‐level overview of the model, in which we include only the most important stocks (rectangles) and the most important feedback loops (small circles/loops). Stocks reflect accumulations, such as the accumulation of infectious people in different stages of the pandemic. Flows represent transitions across stages. To represent flows from one stock to the next, we use double arrows, whereas the single arrows indicate cause‐and‐effect relationships. Delays between cause and effect are shown with a short double line passing through the arrow perpendicularly. Mathematically, each stock is the integral of the flows in, less the flows out. The COVID‐19 transmission subsystem is in the center of Figure 1. We depict the policy interventions as one stock at the top and the diagnostic test supply chain at the bottom.

This COVID‐19 transmission subsystem (see Appendix 1 in the Supporting Information for a full description) reflects the susceptible, infectious, recovered (SIR) epidemic model (Sterman, 2000), which has long‐informed health modeling. The core SIR model continues to be in use (Darabi & Hosseinichimeh, 2020). Figure 1 shows this model in which the infectious population is divided into an undiagnosed (not tested yet) population and a diagnosed (tested) population. We have extended the SIR model with additional stocks (such as asymptomatic and pre‐symptomatic) to represent the transmission of COVID‐19, as well as stages between infection and recovery: quarantine, isolation, hospital, and ICU. Not everyone recovers from COVID‐19, so we also add a stock for the deceased population (to keep Figure 1 readable, we have not shown these additional stocks in Figure 1, but they are explained in Appendix 1 in the Supporting Information). The asymptomatic population can still infect others but does not experience symptoms, so they are unlikely to be tested. The pre‐symptomatic population experiences symptoms after some time and is likely to enter quarantine voluntarily (e.g., due to a runny nose or fever). However, not everyone in this voluntary state follows the rules and stays at home, which may lead to new infections. During quarantine, people can become so sick that they need hospitalization, at which point they get tested (i.e., after getting sick). If there are sufficient diagnostic tests available, people can get tested before experiencing symptoms (i.e., before getting sick), and a positive result sends them into isolation. We anticipate that people take greater care when they are under isolation, as opposed to quarantine, because isolation follows a positive test, whereas quarantine follows just suspicion (Rahmandad et al., 2021). Therefore, we assume that people in isolation do not infect others (as opposed to people in quarantine); the same assumption applies to people in hospitals and ICUs. In practice, they still can infect others, but because it is obvious they are infected, they are less likely to do so. That is, we regard infection by people in isolation, hospitals, or ICUs as nonexistent or at least negligible. Similar to people in quarantine, isolated people can become so sick that they need to be hospitalized. Including hospitalized populations in the model is necessary because, in most countries, the need to flatten the infection curve intensified in the face of capacity shortages in hospitals and ICUs. Therefore, it is important to simulate the impact of different scenarios on capacity requirements, which reflect the curves of infectious populations in hospitals and ICUs. We simplified the model by assuming that people only go to the ICU after being hospitalized first, and people can only die after having spent some time in the ICU.

The most important feedback loop in this COVID‐19 transmission subsystem is a reinforcing one: *contagion* (depicted with a capital R in a small loop in Figure 1). Contagion is what makes people flow from the susceptible stock to the undiagnosed infectious population stocks. After receiving a positive test result, they flow into the diagnosed infectious population. The undiagnosed infectious population can infect others, as indicated by the black single arrow pointing from this stock to the infection rate in Figure 1. This contagion loop is reinforcing because, with more infected people, others can become infected. Figure 1 shows only one reinforcing loop, but our model includes a family of reinforcing contagion loops that go from various stocks of infected people (like asymptomatic and pre‐symptomatic) back to the infection rate. When infection curves rise, the reproduction rate of the disease (i.e., the expected number of cases directly generated by one case) is greater than 1, and the system is in its contagion phase. The infection curve features exponential growth. Growth can be slowed or stopped naturally if there is no one left to infect (i.e., everyone is infected or recovered; the stock of the susceptible population is depleted). At this point, the reproduction rate falls below 1, and the system enters a recovery phase, with declining numbers of new infections. The reinforcing loop now becomes virtuous. In this situation, the balancing loops of depletion and recovery determine the behavior of the system, and the infectious population slowly decreases. After some delay, this also means fewer patients in hospitals and ICUs. The reinforcing loop can also be slowed down if more people are tested (so they know when to isolate) or if policy interventions such as lockdowns reduce the possibility of infection. These interventions will be described next.

The second subsystem refers to the ordering, production, and supply of COVID‐19 diagnostic tests.^1^ We adopt a generic stock management structure (Sterman, 2000), in which the order rate of diagnostic tests depends on an inventory correction (difference between desired and actual inventory level) and actual usage rate (see Appendix 2 in the Supporting Information for a full description; Figure 1 only captures the essence). The desired inventory level is based on policymakers’ forecast of the demand (need for diagnostic tests), and this forecast again depends on policymakers’ expectations about the future number of infections. This expectation is based on the infection rate and on the effect of policy interventions like lockdowns. To account for ordering and delivery delays, the diagnostic test supply chain subsystem consists of three stocks: raw material inventory at the supplier, final inventory at the supplier, and final inventory in a central warehouse in Norway. Connecting this supply chain with COVID‐19 transmission results in a balancing loop: *supply chain interventions to flatten the curve*. This loop is balancing because it stabilizes the system and can dampen growth in new infections. More infections lead to high order rates for diagnostic tests that, after some delay, will increase the final inventory in Norway. This increase then has positive effects on test rates and should reduce the likelihood that infected people infect others (because they know to isolate). Consequently, when infection rates decrease, fewer diagnostic tests will be ordered.

The third subsystem models public policy interventions, including lockdowns and reminding people to focus on hygiene (see Appendix 3 in the Supporting Information for a full description). We model these interventions using stocks (that can have values between 0 and 1) that influence contact rates among people and disease infectivity, which in turn influence new infections. Both the contact rate and infectivity are part of the original SIR model, but in our version, their values depend on public policy interventions. For example, stricter lockdowns reduce the contact rate. We have used the cumulative number of infections to determine the start day of a policy intervention (such as a lockdown) in the first wave and the daily infection rate to determine the end day of the intervention in the first wave and the start and end days of interventions in the second wave. In the first wave, while there was still much uncertainty about the transmission, Norway decided to lock down society relatively early, compared to other European countries (NOS, 2021; Pollock & Steen, 2021). As real infection rates during these early days were unstable (also because of test shortages), it is more likely that policymakers looked at the cumulative number of infections to determine their policies at the beginning of the pandemic. In the second wave, more knowledge about the relationship between the infection rate and hospitalization was available, and policymakers focused more on daily infection rates to determine the need for lockdowns to restrict future hospitalizations.

We model these interventions depending on whether the cumulative number of infections or the daily number of infections reaches a threshold, so the interventions are endogenous. With more infections, a lockdown starts earlier, for example, and if it takes longer to flatten the infection curve, the lockdown will end later. Combining policy interventions and COVID‐19 transmission results in another balancing loop: *policy interventions to flatten the curve*. This loop tries to stabilize the system: When more people test positive, more policy interventions are deployed to bring down the contact rate and infectivity. This action should reduce the number of new infections (flattening curves of new infections and hospitalizations), ultimately creating possibilities to remove lockdowns and reopen society.

### 3.3 Data collection and model calibration

We used actual data from Norway detailing the daily numbers of people infected, deceased, and hospitalized (both normal beds and beds in the ICU) for the model calibration and to identify values of exogenous variables for which no real information is available (e.g., forecast adjustment time by the diagnostic test supplier, delays in opening society). The values of the COVID‐19 transmission parameters came from publications issued by the Norwegian Institute of Public Health, which indicate average infectivity time, average quarantine time, percentage of infected people who develop symptoms, average ICU time, and target test percentage. We could not gain access to real data about diagnostic test inventory levels, ordering policies, or production lead times, so we compared the simulated inventory gap with statements in public reports regarding shortages and surpluses. From this comparison, we concluded that the diagnostic test supply chain subsystem faithfully reproduces the behavior of interest because our simulated periods of shortages and surpluses overlap with the timelines for shortages and surpluses revealed in the public reports. The calibration process and the data we used for calibration and visual inspection are detailed in Appendix 4 in the Supporting Information.

**FIGURE 2:**
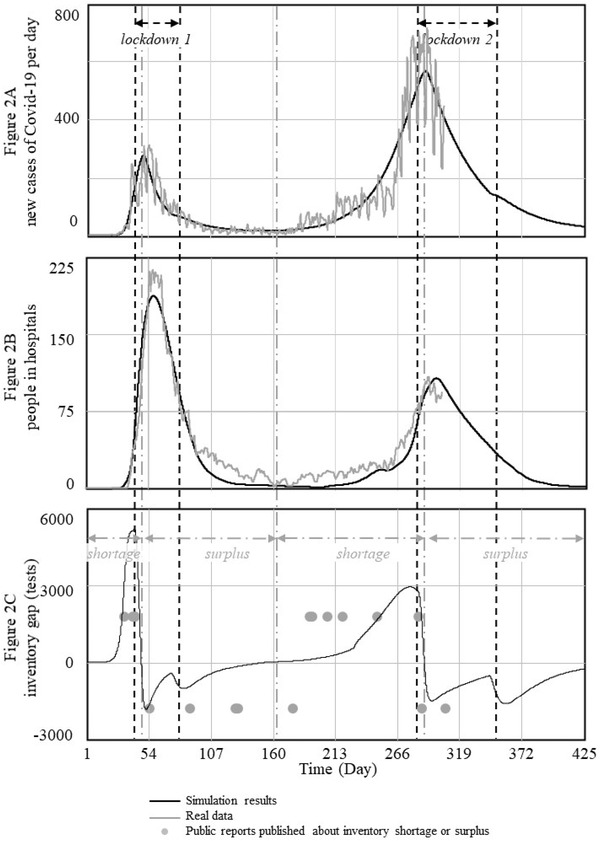
Simulation results and real data of COVID‐19 transmission in Norway (base case)

Figure 2 contains the base case simulation results for daily new cases of COVID‐19 in Norway (Panel A), the number of people in hospitals (Panel B), and the diagnostic test inventory gap (Panel C). It also depicts real data (light gray). Day 1 of our simulation corresponds with February 1, 2020. The vertical, black, dashed lines reference the real dates of the partial lockdown of Norway during the first and second waves. That is, the first partial lockdown started on March 12, 2020 (Day 41; Regjeringen, 2021). Norway then gradually opened society starting on April 19, 2020 (Day 79), but a second partial lockdown started on November 9, 2020 (Day 283). The end of the second lockdown is hypothetical, as it was postponed in Norway due to the outbreak of the Alpha virus mutation, which is excluded from our simulation. However, our model predicts that without this mutation, Norway would have been able to open up gradually starting on day 350 (January 15, 2021); this date is derived endogenously because it depends on the decreasing rate of new infections. The end of the first lockdown is modeled in the same way. Appendix 3 in the Supporting Information provides all the equations we used to calculate the start and end days for lockdowns.

In Figure 2, the vertical, gray, dot‐dashed lines separate periods of diagnostic test shortages from periods of surpluses, according to our simulation (we use the term “test shortage” to denote a lack of available diagnostic tests). We did not get access to real data on test shortages and surpluses, but we use newspaper articles and reports from health organizations to check our simulation results. Gray dots in the lower graph (at the level of 2000 and −2000 tests) indicate whether we find reports about shortages or surpluses. References to these reports are also presented in Appendix 4 in the Supporting Information.

The base case in Figure 2 shows that Norway was relatively early to lock down during the first wave (Pollock & Steen, 2021). The number of cases per day had just reached 200 when the lockdown started. In the second wave, the lockdown occurred much later, when the number of new cases per day was approximately 700. However, the second wave hit younger people harder, and the number of hospitalizations was smaller among this population. Periods of diagnostic test shortages and surpluses overlap with the waves of the pandemic. During the upturn, when the number of infections was increasing exponentially, Norway experienced shortages in both waves. This was caused by the difficulty in forecasting diagnostic test requirements with only partial information and by supply chain delays that are longer than the rapid exponential growth in the number of infections. During downturns, when the number of infections was decreasing again, inventory levels were more than sufficient, again in both waves. Here, imperfect partial information, difficulties in forecasting, and supply chain delays led to excess stocks. Thus, diagnostic test inventory levels behave exactly opposite to what is desired: a shortage when they are needed most, and transmission is out of control, but a surplus when the situation is under control.

### 3.4 Scenario definition

Although Norway never experienced overcrowded hospitals, high death rates, or mass graves, hospital and ICU capacity were stretched, and other patients had to be de‐prioritized. Any intervention to flatten the curve would have been welcome. During the contagion phase, a small intervention may have a large impact because of the typical exponential growth in the number of infections. During the recovery phase, the same intervention may not have such a major impact due to the self‐stabilizing behavior of the system.

The base case scenario reveals that the waves of diagnostic test shortages and surpluses behave countercyclically: When the infection curve is increasing and tests are needed most, inventory is insufficient; when the infection curve is decreasing, inventory is more than sufficient. This behavior motivates our scenario definition, such that we model three situations, with five scenarios each:

1. Reducing diagnostic test shortages as soon as they occur: In the first set of scenarios, Norway receives extra tests from a different country any time it experiences a shortage. In modeling terms, an extra inflow is added to the stock “final inventory of diagnostic tests in Norway” (this stock is shown in Figure 1). This inflow is equal to a percentage of the inventory gap, any time the gap is greater than 0 (i.e., a shortage). For example, the scenario “+100%” means that when the inventory gap is 1000 tests on a particular day, Norway receives 1000 extra tests. We assume the tests come from another country, which can provide help very quickly, so no supply chain delays with respect to ordering materials and producing tests arise, and instead fast shipments of excess stocks from other countries to Norway (we assume that this takes on average 1 day). In the “+20%” scenario, Norway would receive only 200 extra tests when its gap is 1000. We run five scenarios to simulate what would happen if 20%, 40%, 60%, 80%, or 100% of the inventory shortage are covered by a different country that ships tests to Norway. If it took them when needed, without giving anything in return, Norway would be in a competitive situation: *Take what is needed*, without worrying about supplying other countries.
2. Reducing diagnostic test surpluses as soon as they occur: The second set of scenarios simulates donations to a different country whenever Norway experiences a surplus. In modeling terms, it is an extra outflow from the stock “final inventory of diagnostic tests in Norway,” equal to a percentage of the inventory gap when it falls below 0 (i.e., a surplus). For example, the scenario “−100%” implies that when the inventory gap is −1000 tests on a particular day, Norway donates 1000 tests to a different country. Here again, we assume that Norway can ship its excess stocks quickly (we assumed that this takes 1 day on average). We have also run five scenarios again, simulating donations of 20%, 40%, 60%, 80%, and 100% of the inventory surplus. In giving to others and not asking for anything in return, Norway would be engaged in one‐sided collaboration: *Give if there is excess stock to give*.
3. Reducing diagnostic test shortages and surpluses as soon as they occur: Finally, we imagine combining acceptance of tests from a different country when needed (shortage) and donating to a different country if possible (surplus). The simulation model then depicts the stock “final inventory of diagnostic tests in Norway” with a new inflow and new outflow. With the scenario “±100%,” an inventory gap of +1000 tests on a particular day means that Norway accepts 1000 tests from a different country, but if the inventory gap becomes −500 on another day, Norway donates 500 tests to a different country. Again, we run five scenarios, simulating acceptance/donation of 20%, 40%, 60%, 80%, and 100% of the inventory shortage/surplus. Featuring both taking and giving, this represents two‐sided collaboration: *Take what you need, and give when you can*.

Including the base case, we simulate and analyze 16 scenarios, and we discuss the results below.

## 4 SIMULATION RESULTS

Table 1 presents the results for the 16 scenarios, pertaining to some key variables in each of the three model subsystems. Starting with the COVID‐19 transmission subsystem, these key variables are the total number of COVID‐19 cases, the maximum number of patients in hospitals (which is more important than the total because of limited hospital capacity), and the total number of deaths. For the policy intervention subsystem, the most important point is the number of days society was in lockdown. Finally, the key variables in the diagnostic test supply chain subsystem are the total number of tests shipped to Norway, the extra tests accepted by Norway to reduce test shortages, and those donated by Norway that reduce its test surpluses. We present the results for Wave 1, Wave 2, and the total pandemic (Wave 1 + Wave 2). However, this distinction is not necessary for the maximum number of patients in the hospital because the maximum is consistently reached in Wave 1. To facilitate comparisons for each scenario, we also include a column that depicts its relative differences with the base case.

**TABLE 1:** Simulation results of the base case and diagnostic test inventory scenarios

	COVID‐19 transmission										Policy interventions			
	Cases in Wave 1 (people)	Cases in Wave 2 (people)	Total cases (people)		Cases in hospital (people)		Deaths in Wave 1 (people)	Deaths in Wave 2 (people)	Total deaths (people)		Lockdown Wave 1 (days)	Lockdown Wave 2 (days)	Total lockdown (days)	
Scenario	Total	Total	Total	*Rel*.	Max	*Rel*.	Total	Total	Total	*Rel*.	Total	Total	Total	*Rel*.
Base case	9100	44,640	53,740	*1.00*	187	*1.00*	256	243	499	*1.00*	38	67	105	*1.00*
+20% of shortage	8323	44,974	53,297	*0.99*	157	*0.84*	216	239	455	*0.91*	31	64	96	*0.91*
+40% of shortage	7811	45,347	53,158	*0.99*	140	*0.75*	193	234	426	*0.85*	30	65	95	*0.90*
+60% of shortage	7424	45,612	53,036	*0.99*	128	*0.68*	176	229	405	*0.81*	28	64	92	*0.88*
+80% of shortage	7200	45,791	52,991	*0.99*	120	*0.64*	166	225	391	*0.78*	27	66	93	*0.89*
+100% of shortage	6980	45,942	52,923	*0.98*	113	*0.60*	156	221	378	*0.76*	26	67	93	*0.89*
−20% of surplus	8986	44,231	53,217	*0.99*	188	*1.00*	256	249	505	*1.01*	36	64	100	*0.95*
−40% of surplus	8905	43,950	52,855	*0.98*	188	*1.00*	256	251	507	*1.02*	35	62	97	*0.92*
−60% of surplus	8841	43,759	52,600	*0.98*	188	*1.00*	256	252	508	*1.02*	35	60	95	*0.90*
−80% of surplus	8790	43,606	52,396	*0.97*	188	*1.00*	256	253	509	*1.02*	34	60	94	*0.89*
−100% of surplus	8747	43,486	52,233	*0.97*	188	*1.00*	257	253	510	*1.02*	34	59	93	*0.89*
±20% of short./surpl.	8204	44,668	52,872	*0.98*	157	*0.84*	216	242	458	*0.92*	31	63	94	*0.89*
±40% of short./surpl.	7637	44,784	52,421	*0.98*	140	*0.75*	193	238	431	*0.86*	29	63	92	*0.87*
±60% of short./surpl.	7219	44,874	52,093	*0.97*	128	*0.68*	176	234	411	*0.82*	27	63	91	*0.86*
±80% of short./surpl.	6974	44,916	51,891	*0.97*	120	*0.64*	166	232	398	*0.80*	26	64	90	*0.86*
±100% of short./surpl.	6738	44,954	51,692	*0.96*	113	*0.61*	157	229	385	*0.77*	25	64	89	*0.85*

	Diagnostic test supply chain subsystem									
	Tests shipped to NO Wave 1 (tests)	Tests shipped to NO Wave 2 (tests)	Total tests shipped to Norway (tests)		Extra tests accepted Wave 1 (tests)	Extra tests accepted Wave 2 (tests)	Total extra tests accepted (tests)	Excess Tests donated Wave 1 (tests)	Excess tests donated Wave 2 (tests)	Total excess tests donated (tests)
Scenario	Total	Total	Total	*Rel*.	Total	Total	Total	Total	Total	Total
Base case	570,690	2,795,420	3,366,110	*1.00*	0	0	0	0	0	0
+20% of shortage	522,250	2,816,050	3,338,300	*0.99*	10,141	21,401	31,542			
+40% of shortage	490,251	2,839,359	3,329,610	*0.99*	15,859	36,504	52,363			
+60% of shortage	466,055	2,855,945	3,322,000	*0.99*	19,339	47,670	67,009			
+80% of shortage	452,050	2,867,160	3,319,210	*0.99*	21,891	56,297	78,188			
+100% of shortage	437,650	2,877,290	3,314,940	*0.98*	23,517	63,152	86,669			
−20% of surplus	563,624	2,769,706	3,333,330	*0.99*				9844	20,517	30,361
−40% of surplus	558,591	2,752,009	3,310,600	*0.98*				16,765	35,018	51,783
−60% of surplus	554,604	2,739,986	3,294,590	*0.98*				21,916	45,880	67,797
−80% of surplus	551,459	2,730,371	3,281,830	*0.97*				25,901	54,298	80199
−100% of surplus	548,759	2,722,871	3,271,630	*0.97*				29,090	61,012	90,102
±20% of short./surpl.	514,816	2,796,844	3,311,660	*0.98*	10,142	21,349	31,491	8099	20,239	28,337
±40% of short./surpl.	479,371	2,804,069	3,283,440	*0.98*	15,864	36,309	52,173	12,287	34,382	46,669
±60% of short./surpl.	453,220	2,809,720	3,262,940	*0.97*	19,347	47,390	66,737	14,597	44,803	59,400
±80% of short./surpl.	437,936	2,812,354	3,250,290	*0.97*	21,904	55,933	77,837	16,227	52,819	69,046
±100% of short./surpl.	423,179	2,814,701	3,237,880	*0.96*	23,537	62,666	86,203	17,101	59,113	76,214

*Note*: Rel. = relative to the base case, calculated as a ratio: x/y. max = maximum number during the entire simulation. +xx% of shortage = Norway always accepts xx% of its daily diagnostic test inventory shortage from another country. −xx% of surplus = Norway always donates xx% of its daily diagnostic test inventory surplus to another country. ±xx% of short./surpl. = Norway always accepts/donates xx% of its daily diagnostic test inventory shortage from/surplus to another country.

**FIGURE 3:**
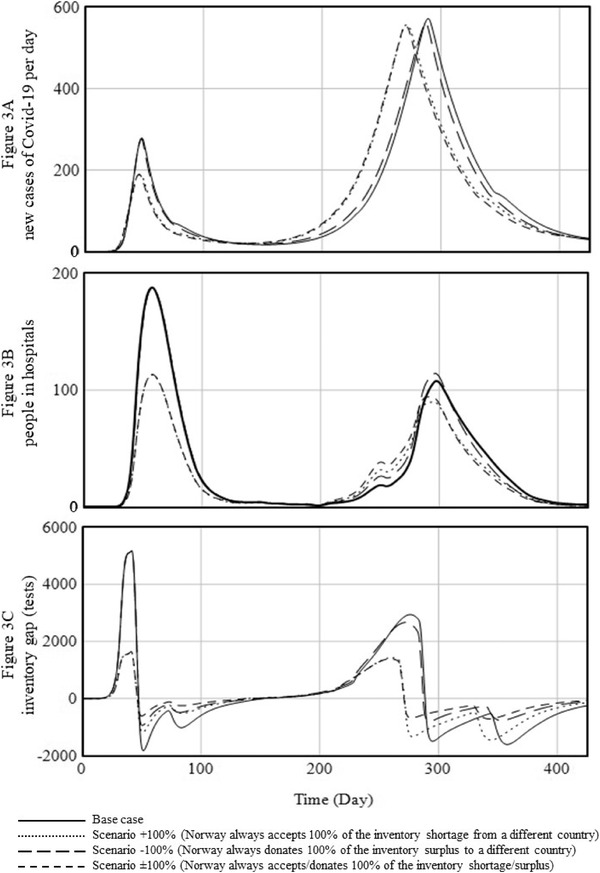
Simulation results of the base case and three scenarios

To complement these numerical results, we depict behaviors over time for the base case and three extreme scenarios (+100%, −100%, and ±100%) in Figure 3. Panel A reveals new cases of COVID‐19 per day, Panel B displays the population in hospitals, and Panel C shows the inventory gap over time. Notably, it is difficult to separate the four graphs in Panels A and B because the behaviors of the base case and the −100% scenario are quite similar as are those of the +100% and ±100% scenarios. The differences are detailed in the total and relative values in Table 1. We specify the simulation results in greater depth next.

### 4.1 Take what you need (competition)

By taking what it needs, Norway would reduce shortages as soon as they occur. All five scenarios that simulate shortage reductions perform better than the base case. Having access to more diagnostic tests when they are needed most (upturn of the wave) flattens the curve, especially during the first wave. In the +100% scenario, the total number of COVID‐19 cases decreases by 2%, hospitalizations by 40%, and the number of deaths by 24%, compared with the base case. Furthermore, the lockdown period would be shortened by 11%. In this +100% scenario, Norway receives almost 87,000 diagnostic tests from a different country, yet the total number shipped to Norway during Waves 1 and 2 would be 2% lower than the base case. This outcome highlights the importance of having sufficient supply when needed (when the infection curve is increasing). Extra supply during the upturn or contagion phase helps flatten the curve, which reduces the future need for diagnostic tests and the number required in total over the entire lifetime of the disease.

These results also exemplify the dynamic behavior and interconnectedness of the three subsystems: Having extra diagnostic tests at the right point in time changes the dynamics of the COVID‐19 transmission in such a way that not only the is curve flattened but policy interventions also can be released (shorter lockdowns), and due to the flattened curve, fewer tests are required in the future. The results of Table 1 also reveal that reducing test shortages help more in Wave 1 than in Wave 2, again due to the interconnectedness of the three subsystems. In Section 3.3, we discussed that Norway's first lockdown happened relatively early in Wave 1 and relatively late in Wave 2. The threshold for locking down society was higher in Wave 2 than in Wave 1, such that the exponential growth of new infections was much higher in Wave 2, when the second lockdown started. Our simulation results show that having extra diagnostic tests *without* an early lockdown flattens the curve somewhat in Wave 2, but not as much as in Wave 1, when extra supply would have been used *with* an early lockdown. For example, in Wave 1, we find 9100 cases in the base case, compared with 6980 cases in the +100% scenario (23% reduction). In Wave 2, however, the base case features 44,640 cases, compared with 45,942 cases in the +100% scenario (3% increase). This difference between Wave 1 and Wave 2 shows that when the reinforcing loop of contagion determines the behavior (infection rates increase rapidly), having access to diagnostic tests alone is insufficient to flatten the curve. Policy interventions are also required. That is why these five scenarios in which Norway receives tests from other countries help more in Wave 1, when there was a low threshold for lockdowns, and consequently an early lockdown, than in Wave 2 (high threshold and late lockdown). In Appendix 5.2.5, we provide more evidence on this statement by simulating additional scenarios with respect to thresholds for lockdowns.

### 4.2 Give when you can (one‐sided collaboration)

By reducing test surpluses as soon as they occur, by donating any excess inventory to other countries, Norway would not have suffered any ill effects relative to the base case. In the −100% scenario, it would have given away more than 90,000 tests, but the curves of its COVID‐19 cases and hospitalizations would not have intensified, and the total number of deaths would remain similar to the base case (2% increase). Surprisingly, the lockdown even appears slightly shorter (105 days in the base case, 93 days in the −100% scenario), mostly due to an earlier end of both lockdowns, caused by an underestimation of the number of infections. That is, when excess diagnostic tests are donated, they cannot be used to test people who have not yet developed symptoms; this could lead to an underestimation of the infection rate, which could again shorten the lockdown period. Shortening the lockdown by a few days does not have significant negative side effects as the balancing recovery loop is already determining the system's behavior.

Considering the importance of reducing test shortages and having sufficient inventory, these findings might seem somewhat counterintuitive. However, the lack of impact of giving away reflects the importance of timing: Diagnostic tests are donated in the downturn, when the exponential growth of infections is already curbed, and the balancing loops dominate the behavior of the entire system. The negative impact of donating tests is also limited because we assume that donations only occur with an inventory surplus. Norway does not donate tests if it needs them, but when it does donate them to another country, global infection rates are impacted.

### 4.3 Take what you need and give when you can (two‐sided collaboration)

Finally, we simulate reducing both test shortages and surpluses as soon as they occur by accepting extra diagnostic tests when needed and donating them when possible. The two‐sided collaboration scenario results are similar to those we obtained from the competitive scenarios, in which Norway only accepted diagnostic tests when needed but did not donate with a surplus. For example, in the ±100% scenario, the total number of COVID‐19 cases drops by 4%, the maximum number of patients in hospitals drops by 39%, and the total number of deaths falls by 23%. The length of the lockdown period is shortened by 15%. The number of diagnostic tests accepted is slightly greater than the number donated: Norway would accept a little more than 86,000 tests from other countries but could only donate approximately 76,000. By receiving help from other countries, its transmission curve flattens to such an extent that its future order rates decrease, so it has fewer surpluses to donate to other countries. Nevertheless, these simulation results show that it would not hurt Norway to donate these 76,000 tests, nor does giving excess tests away hinder the performance of the overall system. This scenario could help other countries in need, and the two‐sided collaboration would facilitate exchanges as it is more equitable.

Figure 3 shows the behavior over time of three situations in which 100% of the inventory shortage is accepted and/or 100% of the inventory surplus is donated. The results in Table 1 show that the +20% and ±20% scenarios already lead to improvements. The largest improvement comes from no collaboration (base case) to the ±20% scenario. For example, hospital occupancy is reduced by 16%, and deaths are reduced by 8% in the ±20% scenario. Increasing to the ±40% scenario yields an additional reduction of 9% in hospital occupancy, compared to the base case and another 6% reduction in deaths. Therefore, the benefits of increasing collaboration are not linear: the ±40% scenario is not twice as good as the ±20% scenario; likewise, the ±80% scenario is not twice as good as the ±40% scenario. This means that when a ±100% scenario sounds too risky, policymakers can already reap many benefits with lower levels of two‐sided collaboration.

### 4.4 Additional analyses

In addition to running the 16 scenarios described above, we performed additional analyses to evaluate the robustness of our model and the sensitivity of our results and recommendations. The robustness checks are described in detail in Appendix 5.1 of the Supporting Information. The sensitivity analyses are described in Appendix 5.2. Here, we only summarize the results of these additional analyses. We performed four types of robustness checks with our model. First, we checked our model using different time steps. Second, we changed the integration methods to evaluate whether these methods influence our results. Third, we checked whether our model can handle a large increase in the percentage of people who need to be hospitalized (like an extreme condition). Fourth and finally, we checked to see whether the model is able to handle the introduction of a mutant virus that rapidly increases the infectivity of COVID‐19, and if so, how this impacts the results. The results of these robustness checks give us confidence that our model is indeed robust to changes in time steps, integration methods, and some extreme situations like more hospitalizations and mutations.

We also performed six sensitivity analyses with our model. The first three analyses explored the impact of changes in typical supply chain parameters: the average shipment time of diagnostic tests from the factory to Norway, the time it takes to reallocate or redistribute diagnostic tests between countries, and the benefits of having (and using) a prepositioned stock tests in Norway. Our sensitivity analyses reveal that shorter shipment times are obviously better, but they cannot compete with two‐sided collaboration. This suggests that although (more) local production (which often reduces shipment times) is good, collaboration may be better. We assumed that diagnostic tests can be reallocated between Norway and other countries within an average of 1 day. We also tested what would happen to two‐sided collaboration when this would take up to 20 days. It is not surprising that the more time it takes to reallocate diagnostic tests, the smaller its positive effect is, but even with a 20‐day delay, the two‐sided collaboration scenario outperforms our base case. We also modeled a prepositioned (or safety) stock in Norway, consisting of 50,000 diagnostic tests. Having such a stock and using it as soon as the first inventory shortage is noticed is a promising intervention. However, this means that policymakers know beforehand what kind of diagnostic tests they need for the next pandemic. The fourth sensitivity analysis explore the effects of increasing the percentage of the population that develops symptoms. We assumed a value of 40%. Increasing this percentage means that more people will develop symptoms, and as such, the “visible” number of infections increases. This does not mean that more people are infected, but simply that fewer people will be asymptomatic. In our fifth sensitivity analysis, we examined the impact of an earlier versus later lockdown in the first wave. This analysis shows that locking down society 1 week earlier has enormous beneficial effects for all subsystems in our model: shorter lockdowns, fewer infected people, less pressure in hospitals, fewer deaths, and fewer diagnostic tests required. An early lockdown seems to perform better than our scenario with two‐sided collaboration. This suggests that when countries cannot collaborate with others or have problems getting access to diagnostic tests, they should implement policy interventions early on, as good and timely interventions can compensate for a lack of tests. Finally, the sixth sensitivity analysis explored the impact of increasing the number of diagnostic tests required per person. As this number influences both the demand, and consequently, the order rate and the shipment rate of diagnostic tests to Norway, this does not influence our results.

## 5 DISCUSSION

We applied an integrated system approach to analyze the interactions of three subsystems: COVID‐19 transmission, diagnostic test supply chain management, and policy interventions. Modeling infectious diseases, supply chain management, or policy interventions is not new, but combining these three subsystems in one single model is rare (Araz et al., 2020) and even goes beyond studies by Duintjer Tebbens and Thompson (2018) and calls by Paul and Venkateswaran (2020) to integrate a disease‐specific medicine supply chain with an epidemic model. Nevertheless, the combination is necessary in pandemics because policymaking, disease transmission expertise, and supply chain competence all need to be combined judiciously to deal with the pandemic. This point represents the key contribution of our paper. Health management systems within and across countries (such as the European Union) are not sufficiently equipped as recent management efforts (e.g., of vaccines) have shown. The dynamic behavior and interconnectedness of the three subsystems have also been insufficiently acknowledged by policymakers or populations. The world was caught by surprise, and the lack of preparedness led to the rapid increase in infections (exponential growth). Such knowledge gaps must be resolved through system designs to deal with future outbreaks and pandemics, efforts that cannot be achieved by policymakers, medical personnel, or supply chain experts alone. They must join forces to identify, revise, and optimize interconnected, nonlinear (exponential growth), dynamic systems.

With a model that combines these three subsystems, we simulate three situations that we compare with an isolationist base case: a competitive policy (take what you need), a one‐sided collaborative policy (give when you can), and a two‐sided collaborative policy (take what you need, give when you can). The findings show that a take‐what‐you‐need policy helps flatten the curve and reduce hospitalizations and deaths while also shortening lockdowns. It also matches a competitive mindset, focused on taking care of the native country without doing anything for others. Over time, however, the long‐term reputation of a country might suffer if it continually acts only competitively, takes what it needs, and never provides help to others. It might not have access to help from other countries in a future crisis. Our findings also show that a give‐when‐you‐can policy (one‐sided collaboration) does not make COVID‐19 transmission worse than the base case because the country only donates excess diagnostic tests when it does not need them. Despite the similar results to the base case, it may be difficult for policymakers to implement; if test shortages are salient in people's recent memory, public opinion might not favor donations. Furthermore, risk‐averse policymakers might want to avoid being blamed for potential future shortages. Insufficient preparedness and a lack of understanding of the interconnectedness, nonlinearities, and dynamics of the system have put the global community in a situation of fear and self‐protection. Accordingly, we recommend the take‐what‐you‐need and give‐what‐you‐can policies. Our results demonstrate that such two‐sided collaboration is possible, as donating excess stock does not create problems for the donator. Also, two‐sided collaboration is not limited to direct collaborations between stable pairs of countries. When a pandemic hits countries at different times, the country from which diagnostic tests are taken does not have to be the precise one that receives supply later.

It may not be surprising that access to more, or at least enough, diagnostic tests helps limit the transmission of COVID‐19 (Anupindi et al., 2020; Ghaffarzadegan & Rahmandad, 2020; Rahmandad et al., 2021). Struben (2020) showed, with a simulation, that an early ramp‐up of diagnostic testing capacity strongly flattens the curve of infections. However, our finding that donating excess supply does not have a negative impact on the curve is novel and useful, offering a clearer understanding of the overall system of waves of infections, diagnostic test inventories, and lockdowns. Better test sharing so that they can be used where and when they are most needed could greatly improve situations, especially in contexts marked by global shortages but uneven supply and demand in different countries at different times. The development of new mutations in countries with high infection rates demonstrates the need for a global perspective: Giving help to other countries will ultimately also benefit the giver.

We argue for collaboration between countries but acknowledge that neighboring countries may show similar shortage patterns. For instance, Denmark and Norway followed each other quite closely (Ritchie et al., 2020). It may therefore be better to consider collaboration between distant countries within the European Union or with the United States or Asia. Comparing the European Union with the United States shows they had different patterns and still do (Ritchie et al., 2020).

The pandemic is a long‐term challenge, with several waves; at the time of writing, it had already lasted more than our simulated 425 days. Thus, policymakers should take a long‐term view on helping their own citizens (take when you need) but also be generous to citizens of other countries (give when you can). Referring to employee productivity, prior research distinguishes “between self‐sacrificing givers and successful ones [by] the willingness to seek help from others” (Grant, 2013, p. 94), which also applies to our research context. Additional waves will most likely occur in the future, vaccine shortages and surpluses are recurring phenomena, and new pandemics are likely to emerge. By asking for help as needed and giving it as they are able, countries can establish sustainable, helpful patterns of behavior over the long term.

However, we also acknowledge that two‐sided, horizontal collaboration can be difficult to maintain in practice (Basso et al., 2019), hindered by lack of trust and risk aversion (Cruijssen et al., 2007). Our integrated approach highlights the importance of timing and reveals that there is a time to be risk‐averse and a time for risk‐taking. The phases of the pandemic (contagion vs. recovery) and the dominant dynamic behavior in these phases (reinforcing vs. balancing) mean that policymakers can and should adopt different behaviors over time, reflecting dynamic policies. In the contagion phase, they cannot afford to, and neither should they turn down extra diagnostic tests to reduce their inventory shortages. They should take what they can get, even if that appears competitive. In the recovery phase, the policy can shift to giving away excess inventory to other countries in need. While such a collaborative approach may seem risky, our findings clearly show that its impact is limited, with no additional strain being added to the national healthcare system because the peak of infections is already past. On a broader scale, this collaborative action would also help flatten the curve in another country and, therefore, at a global level. Furthermore, helping other countries also reduces the re‐inflow from other nations caused by traveling and may prevent the development of mutations in those nations. For the virus, there is no exogeneity: There is only one planet. Finally, in demonstrating that two‐sided collaboration helps flatten the curve, we emphasize the distinction between the time to take and the time to give, depending on the upturns and downturns of infection curves, which are connected to upturns and downturns in inventory levels. Arguably, focusing on temporal dimensions could reduce the role conflict that often arises when decision‐makers seek to address the competition–collaboration dilemma (Stadtler & Van Wassenhove, 2016). Furthermore, the pandemic is a complex dynamic system, with waves that oscillate with different periods and amplitudes reflecting the current state of the networked, globalized world. Such oscillating waves provide opportunities for sharing stocks around the globe because some countries will be in an upturn while others are in a downturn. This characteristic of the pandemic makes it possible to find countries for collaboration, facilitated by efficient information and governance systems, considerations that are beyond the scope of this paper. However, such issues are particularly important, considering the challenges of the global south in gaining access to diagnostic tests (Abdullahi et al., 2020; Seidu et al., 2020).

## 6 CALL FOR ACTION

Based on our current work, we issue a call for action for future research. First, we demonstrate the benefits of two‐sided collaboration, but a study of *how* to implement or improve collaboration between countries is desperately needed. Building upon Besiou and Van Wassenhove (2015) would be one of several pathways forward; using an umbrella approach combining different methodologies to capture the behavior of the entire system before attempting to optimize subsystems. Involving stakeholders in each step of the research process using engaged scholarship methods (Van de Ven, 2007) and group model building (Andersen et al., 2007) could also help in finding out how to get countries to collaborate. Perhaps the collaboration should start small, with neighboring countries or entities within a union (such as the European Union or the United States). Such research could build on the work of Rodriguez‐Pereira et al. (2021) on collaboration between islands in the Caribbean region to establish an equitable allocation scheme for emergency relief items. The extensive literature on collective action could also be relevant in analyzing how to get stakeholders to collaborate (see e.g., Blondin & Boin, 2020).

Second, our results indicate that smart prepositioning and faster transport will help. This also applies to humanitarian logistics, provided one knows where to find excess inventory and how to ship it to countries in need as fast as possible. This requires governments to gather, analyze, and share better data, not just within their countries but also across countries. Good information (systems) for better transparency and a governance structure for collaboration, both in preparedness and response, are required to exploit our strategy. Research on developing platforms and standards for data management could be performed here. Furthermore, having the ability to bend rules applied in normal times by, for example, switching from a slow rigorous border passing process to waiving some controls in an emergency situation would certainly reduce lead times. One could even prepare for this by defining emergency border crossing procedures and trigger mechanisms that would allow swift switches from routine to emergency modes. Stauffer et al. (2016) discuss this for temporary fleet hubs within a large humanitarian organization. Our study suggests to also consider these measures between countries and regions and across organizations. However, further research is required on this option.

Third, vaccine shortages and equity are currently larger issues than diagnostic test shortages. Future research should focus on the effects of reallocating and sharing vaccines, as well as other equipment for medicines and treatment, between countries. Even just within the European Union, having a number of countries sharing vaccines could have a “portfolio” effect, making vaccination programs more effective by leveraging the fact that the peaks and troughs will differ between countries. Continued research might seek ways to leverage the interconnectedness of the systems instead of trying to reduce them. As our research has shown, the varied timing of COVID‐19 waves around the globe can be an opportunity for the improved allocation of scarce resources. Much more research is needed in this space, especially by leveraging the use of simulation models to support such work. Our findings illustrate the temporal dimension of combining competition and cooperation (Hoffman et al., 2018). Our discussion of sequential coopetition, involving the taking and giving of scarce resources, indicates some similarities with risk pooling through joint stockpiles for disaster relief when demand is diverse because disasters strike at different times in different places (Balcik et al., 2019; Rodriguez‐Pereira et al., 2021; Toyasaki et al., 2017). However, unlike physical stockpiles that require comprehensive risk‐ and cost‐sharing mechanisms, our findings imply alternative risk‐pooling options, such as taking and giving depending on how disasters evolve, which can function as a virtual and flexible mitigation strategy. This is akin to strategic agility, where resources are kept central and allocated only when needed (see e.g., Stauffer et al., 2018). Our paper provides a more dynamic approach in terms of demand compared to Rodriguez‐Pereira et al. (2021) and answers their call for research on the value of incorporating “the implications of the cost of inaction by wealthier countries on the long‐term” (p. 18).

Finally, future research might compare our suggestions with other recommended interventions, such as increasing the number of suppliers, local production, or prepositioning stocks. In Appendices 5.2.1 and 5.2.3, we describe additional scenarios that relate to local production and prepositioned stocks. Interestingly, we found that local production does not seem to outperform our scenario with two‐sided collaboration and that prepositioning only outperforms two‐sided collaboration in specific circumstances. Therefore, analyzing the benefits of such interventions is an interesting avenue for further research.

Although policymakers probably agree that an extra diagnostic test inventory would help, they likely resist the idea of giving away excess inventory. Their risk‐averse behavior leads them to prioritize their own citizens as if they were competing with other countries. This behavior is justified in the contagion phase, but policymakers can be overly careful in the recovery phase. Our model offers an analysis of the actual risk of giving away excess inventory. It is very small, and the benefits for another country that has just entered the contagion phase are huge. Therefore, parochial reactions are not justified. In line with the UN Sustainable Development Guideline to “leave no one behind,” policymakers (and citizens) should think globally and avoid keeping stocks of critical resources when others need them badly. In terms of COVID‐19 diagnostic tests, this insight may have come too late, but it is still relevant for the distribution of vaccines (or new diagnostic tests to detect new mutations). Limited global supplies need to be used in the best possible way, where and when needed, and it is important to find ways to achieve more equitable access.

## Supporting information

Supporting Information

Supporting Information

## ENDNOTE

1 By testing, we refer to molecular‐assay testing, a confirmatory test for detecting active infections, not immunoassay tests for antigens or antibodies, which mainly detect whether a person has been infected previously. Our focus is on testing kits, which are required to establish sufficient testing capacity (Behnam et al., 2020). The emerging variations and developments in testing solutions are not included in our model.
