# Supplementary material for: Collaboration–competition dilemma in flattening the COVID‐19 curve
Source: Prod Oper Manag. 2022 Apr 14:10.1111/poms.13709. Online ahead of print. doi: 10.1111/poms.13709 (PMC9115479; doi:10.1111/poms.13709)
Supplement: Supplementary file 1 — Supporting Information [file POMS-9999-0-s001.docx]

**E-Companion**

**“Collaboration–Competition Dilemma in Flattening the COVID-19 Curve”**

Kim E. van Oorschot, Luk N. van Wassenhove, Marianne Jahre

This e-companion consists of the following material:

1. Model Documentation of the COVID-19 Transmission Subsystem 2
2. Model Documentation of the Diagnostic Test Supply Chain Subsystem 7
3. Model Documentation of the Policy Interventions Subsystem 13
4. Model Calibration 17
5. Robustness and Sensitivity 24
6. Model Assessment Results 47
7. Listing All Variables with Values and Units 48
8. References 51

**Appendix 1. Model Documentation of the COVID-19 Transmission Subsystem**

Figure A below shows the stocks, flows and variables used to model the transmission of COVID-19 (for the purpose of readability and clarity we show only the main variables and their interrelationships). In Figure A, stocks are depicted in boxes, flows are depicted with double arrows with a valve. Values for the blue variables were found through calibrating the model with real data about COVID-19 infections in Norway over time (this will be described in Appendix 4).Values for the blue underlined bold exogenous variables are taken from reports published by the Norwegian Institute of Public Health (<https://www.fhi.no/sv/smittsomme-sykdommer/corona/koronavirus-modellering/>). Green variables link this subsystem to the other two subsystems: the diagnostic test supply chain (discussed in Appendix 2), and the policy interventions (discussed in Appendix 3).

The model is based on the SIR-model described by Sterman (2000). This model describes three stocks. The first stock is a susceptible population that, after infection, becomes an infected population (second stock). When the infection is over, the population flows to the recovered population (third stock). This SIR-model is adapted to reflect the characteristics of COVID-19 in Norway. As a result, we have nine stocks:

- Susceptible Population (*S*): people who may be infected by COVID-19.
- Infectious Asymptomatic Population (*IAP*): infected people who experience no symptoms but can infect other people
- Infectious Pre-symptomatic Population (*IPP*): infected people who experience symptoms and can infect other people
- Infectious Population in Quarantine (*IQ*): infected people who experience symptoms but who do not know whether they have COVID-19 because they have not been tested yet
- Infectious Population in Isolation (*II*): infected people who experience symptoms and know they have COVID-19 after being tested positive
- Infectious Population in Hospital (*IH*): infected people who need hospital care
- Infectious Population in Intensive Care Units (*IIC*): infected people who need intensive care (e.g., treatment with respirator)
- Recovered Population (*R*): people who recovered from COVID-19
- Deceased Population (*DP*): people who deceased due to the COVID-19 infection

**Figure A. Stocks, flows, and variables of the COVID-19 transmission subsystem**

In Table A, the equations to model these nine stocks are explained. We start our simulation on February 1, 2020 (Norway’s first case was discovered on February 21, 2020). We run the simulation until March 31, 2021 (425 days). To calibrate the model, we have used real data from Norway until December 1, 2020 (304 days). This means that new variants, like the more infectious Alpha and Beta variants of COVID-19, are excluded from our model since these variants were not discovered in Norway before December 1, 2020.

**Table A. COVID-19 Transmission Subsystem**

**Formulations and comments Units**

Initial time = 1 Days

The initial time for the simulation (February 1, 2020)

Final time = 425 Days

The final time for the simulation (March 31, 2021)

Time step = 0.0625 Days

The time step for the simulation

These simulation settings (initial time, final time, and time step) apply to all subsystems.

$S\left( t \right)=S\left( 0 \right)+\int_{0}^{t} \left( -air(s)-pir\left( s \right) \right)ds;S\left( 0 \right)=initial S$ people

The susceptible population (*S*) is a stock that starts at the level of the *initial S* and is depleted whenever a person is infected, either via the asymptomatic infection rate (*air*) or the pre-symptomatic infection rate (*pir*).

$air\left( t \right)=\left( 1-pds \right)*effc(t)*effi(t)*S(t)*((IAP(t)+IPP(t)+riIQ*IQ(t))/N(t))$ people/day

$pir\left( t \right)=pds*effc(t)*effi(t)*S(t)*((IAP(t)+IPP(t)+riIQ*IQ(t))/N(t))$ people/day

The asymptomatic (*air*) and pre-symptomatic (*pir*) infection rate have similar equations. The difference between the two is defined by the percentage of the infected population that develops symptoms (*pds*). Besides the susceptible population (*S*), the infection rate is determined by the effective contact rate (*effc*), the effective infectivity (*effi*), the infectious asymptomatic population (*IAP*), the infectious pre-symptomatic population (*IPP*), and the infectious population in quarantine (*IQ*) and the total population (*N*). This equation is similar to the equation used to define the infection rate in the original SIR-model (p. 302, Sterman, 2000). We assume that not everyone in quarantine will be completely careful (people may not stay in quarantine even when they say so; they may still go shopping, etc.). This is expressed by the relative infectivity of the infectious population in quarantine (*riIQ*). The higher this factor, the higher the infection rate coming from people in quarantine. Note, that *effc* and *effi* will be discussed in Appendix 3 when we define policy interventions.

$IAP\left( t \right)=IAP\left( 0 \right)+\int_{0}^{t} \left( air(s)-rra\left( s \right) \right)ds;IAP\left( 0 \right)=0$ people

$rra\left( t \right)=IAP(t)/ait$ people/day

The infectious asymptomatic population (*IAP*) is the integral of the asymptomatic infection rate (*air*) minus the recovery rate of the asymptomatic population (*rra*). This last rate is modeled as a first-order material delay: the infectious asymptomatic population (*IAP*) divided by the average infectivity time (*aift*).

$IPP\left( t \right)=IPP\left( 0 \right)+\int_{0}^{t} \left( pir\left( s \right)-drs\left( s \right)-trbs(s) \right)ds;IPP\left( 0 \right)=initial IPP$ people

$drs\left( t \right)=dtrbs\left( t \right)-trbs(t)$ people/day

$trbs\left( t \right)=MIN(dtrbs\left( t \right),ptrbs\left( t \right))$ people/day

$dtrbs\left( t \right)=IPP(t)/att$ people/day

The pre-symptomatic population (*IPP*) is the integral of the pre-symptomatic infection rate (*pir*) minus the discovery rate of symptoms (*drs*) and the testing rate before symptoms (*trbs*). We assume that symptomatic people can follow two paths: either they discover symptoms naturally, after which they voluntary quarantine themselves (*drs*), or they take a diagnostic test because they suspect that they may be infected even though they do not experience symptoms yet (*trbs*). Ideally, the entire infectious pre-symptomatic population (*IAP*) is tested, which is defined by the desired testing rate before symptoms (*dtrbs*). However, this rate is constrained by the availability of diagnostic tests, which is defined by the possible testing rate before symptoms (*ptrbs*). (Note that *ptrbs* will be defined in Appendix 2.2).

$DP\left( t \right)=DP\left( 0 \right)+\int_{0}^{t} \left( dr(s) \right)ds;DP\left( 0 \right)=0$ people

$dr\left( t \right)=ndp*IIC(t)/aict$ people/day

$N\left( t \right)=initial N-DP(t)$ people

The deceased population (*DP*) is the integral of the death rate (*dr*). We assume that only people who are in intensive care units can die from COVID-19. As such, the death rate depends on the infectious population in intensive care units (*IIC*), a normal death percentage (*ndp*) and an average time on the IC (*aict*). Finally, the deceased population feeds back into the total population (*N*). The total population (N) is equal to the initial total population (initial N) minus the deceased population (DP).

$IIC\left( t \right)=IIC\left( 0 \right)+\int_{0}^{t} \left( aric\left( s \right)-dr\left( s \right)-rric(s) \right)ds;IIC\left( 0 \right)=0$ people

$aric\left( t \right)=pic\left( t \right)*IH(t)/ahtic$ people/day

$rric\left( t \right)=\left( 1-ndp \right)*IIC(t)/aict$ people/day

$IH\left( t \right)=IH\left( 0 \right)+\int_{0}^{t} \left( arhi\left( s \right)+arhq\left( s \right)-aric\left( s \right)-rrh(s) \right)ds;IH\left( 0 \right)=0$ people

$arhi\left( t \right)=ph\left( t \right)*II(t)/aith$ people/day

$arhq\left( t \right)=MIN(ptr,ph\left( t \right)*IQ(t)/aqth$) people/day

$rrh\left( t \right)=(1-pic\left( t \right))*IH(t)/ahtr$ people/day

The infectious population in intensive care units (IIC) is the integral of the admission rate of people coming from a “normal” hospital (*aric*) minus the death rate (*dr*) and the recovery rate (*rric*). We assume here that people can only be admitted to intensive care units when they are already in the hospital. This admission rate to intensive care units is therefore defined by the infectious population in hospitals (*IH*), the percentage of people that needs to go intensive care units (*pic*) and the average time in the hospital before admission to intensive care units (*ahtic*). The infectious population in hospitals (*IH*) is the integral of the sum of two inflows to hospitals minus the sum of two outflows. We assume that people who need to be admitted to hospitals come from either quarantine (*arhq*) or isolation (*arhi*). The first group, coming from a voluntary quarantine, needs to be tested in the hospital (so this group requires diagnostic tests). The second group was already tested before they went in isolation. These two rates are defined by the percentage of the infectious population in either isolation or quarantine that needs to go to the hospital (*ph*) and the average time in isolation (*aith*) or quarantine (*aqth*) before hospitalization. The two outflows of the *IH* are the admission rate to intensive care units (*aric*) and the recovery rate after hospital (*rrh*). This last rate depends on the percentage of people that need to go the IC (*pic*) and the average hospital time before recovery (*ahtr*).

$ph\left( t \right)=lph\left( t \right)*IF THEN ELSE(time\leq e1,mph1,mph2)$ dmnl

$pic\left( t \right)=lph\left( t \right)*IF THEN ELSE(time\leq e1,mpic1,mpic2)$ dmnl

The real data of the infection rate and the number of people admitted to hospitals and intensive care units, revealed that there were differences between the first and second wave. More people needed to go to hospitals and ICUs in the first wave, compared to the second wave. As a result, a fixed percentage for hospitalizations (*ph*) and for admissions to the ICUs (*pic*) for both waves would not work. In the first wave, the percentage of older people that was infected was higher than in the second wave. On the left, in Figure B, the gray line shows the percentage of infected people that is older than 80 years (source: <https://www.vg.no/spesial/corona/>), the black line is based on the same data, but removes outliers to smoothen the curve. This figure shows that during the first wave (from day 1 to day 151, which is defined as the end of wave 1, *e1*), the percentage of infected people older than 80 was higher than after the first wave. It is likely that this also caused a higher percentage of people that had to go to hospitals and intensive care units in this period. As a result, we have defined the *ph* and *pic* as a function over time (lookup) and used the shape of the black line depicted in Figure B as a guideline for finding the right multipliers (constants) for the first (*mph1, mpic1*) and second wave (*mph2, mpic2*) with calibration (discussed in Appendix 4). The resulting values for *ph* after using the multipliers for the first and second wave (*mph1, mph2*) are depicted on the right in Figure B. The *pic* uses the same lookup (black line on the left in Figure B) but with different multipliers for the first and second wave (*mpic1, mpic2*).

$$lph\left( t \right)=\left[ \left( 0,0 \right)-\left( 430,0.1 \right) \right],$$

$(1,0),(30,0.00917431), (58,0.0813758),(72,0.0659722),(219,0.0103021), (317,0.0207603)$ dmnl

For example, on day 58, the *lph* is 0.0814, the *mph1* is 0.151231 (a constant that was derived through calibration, see Appendix 4), leading to a *ph* of 0.0123.


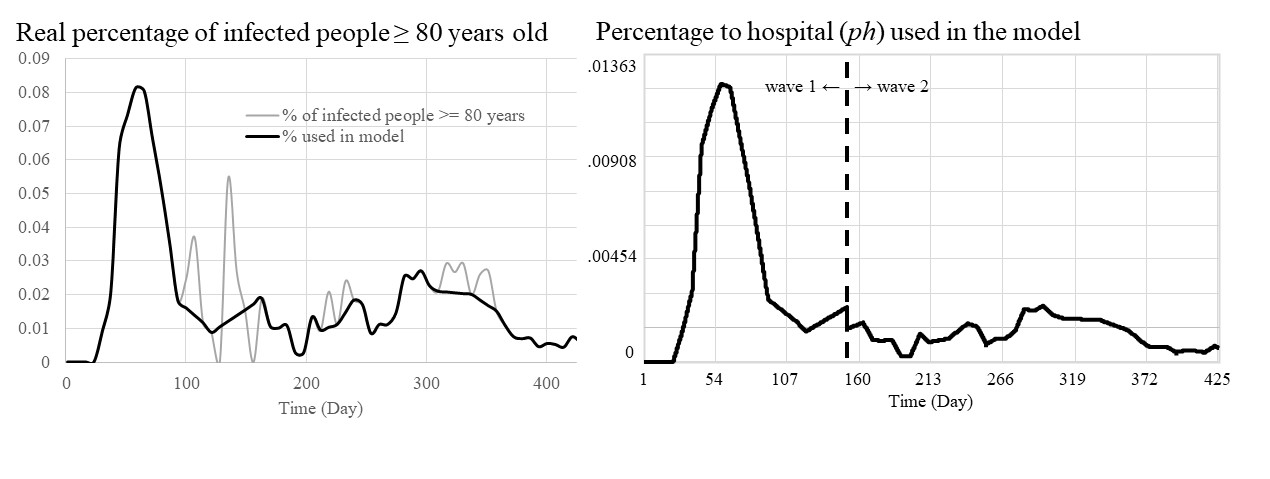


**Figure B. Age differences in infectious population over time**

$II\left( t \right)=II\left( 0 \right)+\int_{0}^{t} \left( trbs\left( s \right)-arhi\left( s \right)-rri(s) \right)ds;II\left( 0 \right)=0$ people

$rri\left( t \right)=(1-ph\left( t \right))*II(t)/ait$ people/day

$IQ\left( t \right)=IQ\left( 0 \right)+\int_{0}^{t} \left( drs\left( s \right)-arhq(s)-rrq\left( s \right) \right)ds;IQ\left( 0 \right)=0$ people

$rrq\left( t \right)=(1-ph\left( t \right))*IQ(t)/aqt$ people/day

$R\left( t \right)=R\left( 0 \right)+\int_{0}^{t} \left( rrh\left( s \right)+rri\left( s \right)+rrq\left( s \right)+rra\left( s \right)+rric(s) \right)ds;R\left( 0 \right)=0$ people

The infectious population in isolation is the integral of the people that are tested positively (testing rate before symptoms, *trbs*) minus the admission rate to hospitals from isolation (*arhi*) and the recovery rate after isolation (*rri*). This recovery rate is defined by the percentage that needs to go to the hospital (*ph*), the infectious population in isolation (*II*) and the average time in isolation (*ait*). The infectious population in quarantine (*IQ*) is the integral of the number of people voluntary quarantining themselves (discovery rate of symptoms, *drs*) minus the recovery rate after quarantine (*rrq*) and the people who need to be hospitalized (admission rate to hospitals from quarantine, *arhq*). The recovery rate after quarantine is defined by the percentage that needs to go to the hospital (*ph*), the infectious population in quarantine (*IQ*) and the average time in quarantine (*aqt*). Finally, the recovered population (*R*), is the integral of the sum of all people recovering during their time in quarantine (*rrq*), isolation (*rri*), hospital (*rrh*), intensive care (*rric*) and the people recovering without even knowing they were infected (*rra*).

**Appendix 2. Model Documentation of the Diagnostic Test Supply Chain Subsystem**

**2.1 Expectations and forecasts**

In Appendix 1, we discussed how we modeled COVID-19 transmission. To be able to test people that have or suspect they have COVID-19, diagnostic tests need to be ordered. The number of diagnostic tests that health policymakers need to order depends on their *expectation* about the COVID-19 transmission. As such, the diagnostic test supply chain subsystem consists of two parts: in the first part, the expected COVID-19 transmission is modeled which leads to forecast of the required diagnostic tests and a desired inventory level of these diagnostic tests. In the second part, the ordering, production, and shipment of these diagnostic tests to Norway is modeled. Figure C below shows the stocks, flows and variables used to model the expected COVID-19 transmission. Values for the blue variables were found through calibrating the model with real data about COVID-19 infections in Norway over time (this will be described in Appendix 4). Green variables link this subsystem to the policy interventions subsystem (described in Appendix 3), and the second part of the diagnostic test supply chain (described in Appendix 2.2).

We do not know how and when health policymakers in Norway actually started modeling COVID-19 transmission, but relatively early in the pandemic (April 15, 2020), we found a publication on the website of the Norwegian Institute of Public Health explaining that a SEIR-model was used to model the infection rate (<https://www.fhi.no/sv/smittsomme-sykdommer/corona/koronavirus-modellering/>). This model divides the population in sustainable, exposed (pre-symptomatic), infectious (both asymptomatic and pre-symptomatic) and recovered. We have used this information to formulate five new stocks that are used to estimate policymakers’ expectations about infections over time: the expected pre-symptomatic infection rate (*epir*). This *epir* is then used to forecast how many diagnostic tests need to be ordered to make sure the final inventory of such diagnostic tests is equal to the desired final inventory. The values of the constants that are used in this SEIR-model are not known, therefore, we have used the same values as in the COVID-19 transmission subsystem for *initial S, initial N, att, aqt, aift,* and *pds.* Two new constants were introduced: the expected relative infectivity of infectious people in quarantine (*eriIQ*) and the expected initial infectious pre-symptomatic population (*eiIPP*). We introduced these two constants because their real counterparts (*riIQ* and *iIPP*) are probably very difficult for policymakers to determine. Therefore, we assumed that the expected values of these constants (*eriIQ* and *eiIPP*) will be slightly different from the real values used in the COVID-19 transmission subsystem. These expected values were found through calibration (which will be discussed in Appendix 4).

**Figure C. Stocks, flows and variables of *expected* COVID-19 transmission and forecast of diagnostic test rate**

**Table B. Diagnostic test Supply Chain Subsystem: Expectations and forecasts**

**Formulations and comments Units**

$ES\left( t \right)=ES\left( 0 \right)+\int_{0}^{t} \left( -eair(s)-epir\left( s \right) \right)ds;S\left( 0 \right)=initial S$ people

The expected susceptible population (*ES*) is a stock that starts at the level of the *initial S* and is depleted whenever a person is infected, either via the expected asymptomatic infection rate (*eair*) or the expected pre-symptomatic infection rate (*epir*).

$eair\left( t \right)=\left( 1-pds \right)*eci(t)*ES(t)*((EIAP(t)+EIPP(t)+eriIQ*EIQ(t))/initial N)$ people/day

$epir\left( t \right)=pds*eci(t)*ES(t)*((EIAP(t)+EIPP(t)+eriIQ*EIQ(t))/initial N)$ people/day

The expected asymptomatic (*eair*) and pre-symptomatic (*epir*) infection rate have similar equations. The difference between the two is defined by the percentage of the infected population that develops symptoms (*pds*). Besides the expected susceptible population (*ES*), the infection rate is determined by the expected contact rate and infectivity (*eci*), the expected infectious asymptomatic population (*EIAP*), the expected infectious pre-symptomatic population (*EIPP*), the expected infectious population in quarantine (*EIQ*), and the total population (assumed equal to the initial total population (*initial N*). This equation is similar to the equation used to define the infection rate in the original SIR-model (p. 302, Sterman, 2000), and as Norwegian health policymakers use a SEIR-model, it is fair to assume that a similar equation is used in their model. We assume that not everyone in quarantine will be completely careful (people may not stay in quarantine even when they say so; they may still go shopping, etc.). This is expressed by the expected relative infectivity of the infectious population in quarantine (*eriIQ*). The higher this factor, the higher the infection rate coming from people in quarantine.

To calculate the expected asymptomatic and pre-symptomatic infection rates, policymakers also have to make estimations about the contact rate and infectivity. Contact rates and infectivity are influenced by policy interventions, like lockdowns, but it is difficult to predict how well these interventions will work. We have therefore used a trend estimation process (p. 635, chapter 16, Sterman 2000):

$eci\left( t \right)=Pci\left( t \right)*(1+PTci\left( t \right))$ dmnl/day

$Pci\left( t \right)=Pci\left( 0 \right)+\int_{0}^{t} cPci\left( s \right)ds;Pci\left( 0 \right)=iPci(0)$ dmnl/day

$iPci\left( 0 \right)=ci(0)$ dmnl/day

$cPci\left( t \right)=(ci(t)-Pci(t))/tPci$ dmnl/day/day

$Rci\left( t \right)=Rci\left( 0 \right)+\int_{0}^{t} cRci(s)ds;Rci\left( 0 \right)=iPci$ dmnl/day

$cRci\left( t \right)=(Pci(t)-Rci(t))/hRci$ dmnl/day/day

$itci\left( t \right)=(Pci\left( t \right)-Rci\left( t \right))/ titci$ dmnl

$PTci\left( t \right)=PTci\left( 0 \right)+\int_{0}^{t} cPTci(s)ds;PTci\left( 0 \right)=0$ dmnl

$cPTci\left( t \right)=(itci(t)-PTci(t))/tPTci$ dmnl/day

In the trend estimation process as defined by the equations above, the expected contact rate and infectivity (*eci*) used by policymakers to predict infection rates is calculated by a combination of the perceived contact rate and infectivity (*Pci*) and the perceived trend in these variables (*PTci*). The *Pci* adapts gradually to the real contact rate and infectivity (*ci*). (We assume that before the pandemic, that is at the start of the simulation, the *Pci* is equal to *ci*.) This variable will be defined in Appendix 3 as this depends on policy interventions. The perceived trend (*PTci*) depends on the ratio of the perceived contact rate and infectivity and a reference contact rate and infectivity (*Rci*); this ratio is called the indicated trend (*itci*).

$FDTR\left( t \right)=FDTR\left( 0 \right)+\int_{0}^{t} cFDTR(s)ds;FDTR\left( 0 \right)=0$ people/day

$cFDTR\left( t \right)=(epir\left( t \right)-FDTR(t))/fat$ people/day/day

$gfdtr\left( t \right)=FDTR\left( t \right)*tpp/ttp$ tests/day

$dFI\left( t \right)=gfdtr\left( t \right)*mic$ tests

To determine how many diagnostic tests to order, a forecast needs to be made about the number of diagnostic tests that will be required per day in the future (*FDTR*). This forecast is based on the expected pre-symptomatic infection rate (*epir*) and a forecast adjustment time (*fat*). For each diagnostic test that is positive, much more diagnostic tests need to be taken (because also a lot of people that get tested do not have COVID-19). This is defined as the target test percentage (*ttp*). (For example, during the first 304 days of the pandemic, Norway had used in total 2293090 diagnostic tests, and the total number of COVID-19 cases were 36631, which leads to a *ttp* of 0.01597.) By dividing the *FDTR* by the *ttp*, we find the gross forecast diagnostic test rate (*gfdtr*). We assume that we only need one diagnostic test per person (*tpp*). Finally, by multiplying the *gfdtr* with the minimum inventory coverage (*mic*) we find the desired final inventory level of diagnostic tests in Norway (*dFI*).

The remaining four stocks in the SEIR-model used by health policymakers in Norway are defined as follows:

$EIAP\left( t \right)=EAPP\left( 0 \right)+\int_{0}^{t} \left( eair\left( s \right)-erra\left( s \right) \right)ds;EAPP\left( 0 \right)=0$ people

$erra\left( t \right)=EIAP(t)/aift$ people/day

$EIPP\left( t \right)=EIPP\left( 0 \right)+\int_{0}^{t} \left( epir\left( s \right)-edrs\left( s \right) \right)ds;EIPP\left( 0 \right)=eiIPP$ people

$edrs\left( t \right)=EIPP(t)/att$ people/day

$EIQ\left( t \right)=EIQ\left( 0 \right)+\int_{0}^{t} \left( edrs\left( s \right)-errq\left( s \right) \right)ds;EIQ\left( 0 \right)=0$ people

$errq\left( t \right)=EIQ(t)/aqt$ people/day

$ER\left( t \right)=ER\left( 0 \right)+\int_{0}^{t} \left( erra\left( s \right)+errq\left( s \right) \right)ds;ER\left( 0 \right)=0$ people

**2.2 Ordering, production and shipment of diagnostic test kits**

Figure D below shows the stocks, flows and variables used to model the COVID-19 diagnostic test supply chain. Values for the blue variables were found through calibrating the model with real data about COVID-19 infections in Norway over time (this will be described in Appendix 4). Green variables link this subsystem to the other subsystem (COVID-19 transmission, Appendix 1) and the first part of the diagnostic test supply chain subsystem (Appendix 2.1). The two pink variables were used to simulate our scenarios (accepting & donating diagnostic tests). For most of the variables in the diagnostic test supply chain we did not have actual data though. We know how many diagnostic tests were used. Furthermore, based on newspaper articles and articles published by health organizations we can distinguish periods of time when inventory was low or high. These articles were used as a rough check of the inventory gap we simulated with our model (also described in Appendix 4). We have assumed a basic stock management policy (Sterman, 2000), not only for Norway (that is managing its own inventory) but also for the “factory” that is purchasing materials required for producing diagnostic tests, producing these diagnostic tests and shipping them to Norway.


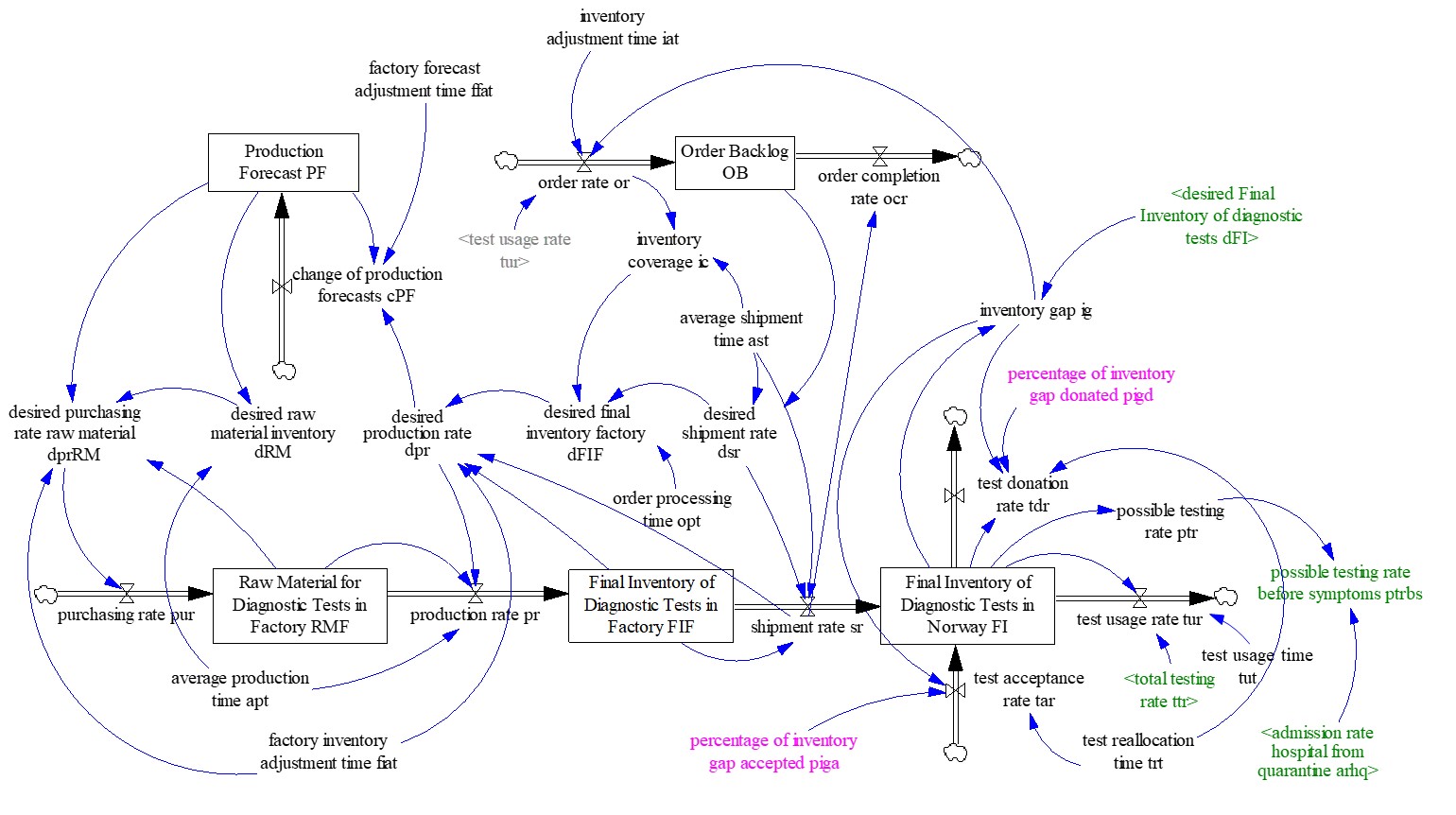


**Figure D. Stocks, flows and variables of the diagnostic test supply chain subsystem**

**Table C. Diagnostic test Supply Chain Subsystem: Ordering, production and shipment**

**Formulations and comments Units**

$ig\left( t \right)=dFI\left( t \right)-FI\left( t \right)$ tests

$or\left( t \right)=MAX(0,tur(t)+ig(t)/iat)$ tests/day

$OB\left( t \right)=OB\left( 0 \right)+\int_{0}^{t} \left( or\left( s \right)-ocr(s) \right)ds;OB\left( 0 \right)=0$ tests

$ocr\left( t \right)=sr(t)$ tests/day

$FI\left( t \right)=FI\left( 0 \right)+\int_{0}^{t} \left( sr\left( s \right)+tar(s)-tur\left( s \right)-tdr\left( s \right) \right)ds;FI\left( 0 \right)=0$ tests

$tar\left( t \right)=piga*MAX(0,ig\left( t \right))/trt$ tests/day

$tdr\left( t \right)=MIN(FI\left( t \right), pigd*-MIN\left( 0,ig\left( t \right) \right))/trt$ tests/day

The desired inventory level of diagnostic tests (*dFI*) is compared with the actual final inventory level of diagnostic tests in Norway (*FI*) to calculate the inventory gap (*ig*). Together with the diagnostic tests used per day (*tur*, described later) and the inventory adjustment time (*iat*), this gap is used to determine the order rate of diagnostic tests from the factory (*or*). The diagnostic test factory collects all incoming orders in the order backlog (*OB*). The backlog is depleted when orders are completed (*ocr*). This order completion rate is equal to the shipment rate (*sr*, described later) of diagnostic tests from the factory to Norway. The final inventory of diagnostic tests in Norway (*FI*) normally has one inflow (shipment rate, *sr*) and one outflow (test usage rate, *tur*). But, when simulating the impact of accepting extra diagnostic tests from a different country or donating diagnostic tests to a different country, an extra in- and outflow are required. The diagnostic test acceptance rate (*tar*) is defined by the inventory gap (*ig*), the time it takes to reallocate diagnostic tests (*trt*) and the percentage of the inventory gap that is accepted from another country (*piga*). Diagnostic tests can only be accepted when there is an inventory shortage (inventory gap is positive). Similarly, the diagnostic test donation rate (*tdr*) is also defined by the inventory gap (*ig*), the time it takes to reallocate diagnostic tests (*trt*) and the percentage of the inventory “gap” that is donated to another country (*pigd*). Note that diagnostic tests can only be donated when the inventory gap is negative, that is, when there is an inventory surplus.

$sr\left( t \right)=MIN(FIF(t)/ast,dsr(t))$ tests/day

$dsr\left( t \right)=OB(t)/ast$ tests/day

$FIF\left( t \right)=FIF\left( 0 \right)+\int_{0}^{t} \left( pr\left( s \right)-sr(s) \right)ds;FIF\left( 0 \right)=0$ tests

$pr\left( t \right)=MIN(RMF(t)/apt,MAX(0,dpr(t)))$ tests/day

$dpr\left( t \right)=sr\left( t \right)+(dFIF(t)-FIF(t))/fiat$ tests/day

$dFIF\left( t \right)=MAX(ic(t),dsr\left( t \right)*opt)$ tests

$ic\left( t \right)=or\left( t \right)*ast$ tests

The factory that produces diagnostic tests for Norway ships these diagnostic tests to Norway based on the minimum of the desired shipment rate (*dsr*) and the final inventory of diagnostic tests in the factory (*FIF*) divided by the average shipment time (*ast*). The desired shipment rate is equal to ratio of the order backlog (*OB*) and the average shipment time (*ast*). The final inventory of the factory (*FIF*) is the integral of the production rate (*pr*) minus the shipment rate (*sr*). This production rate depends on the desired production rate (*dpr*) but could be constrained by the ratio of the inventory of raw material in the factory (*RMF*) and the average production time (*apt*). The desired production rate (*dpr*) depends on the actual shipment rate (*sr*) plus corrections that are required to adjust the final inventory of the factory. The latter depends on the difference between the desired final inventory (*dFIF*) and the current final inventory (*FI*), and the factory inventory adjustment time of the factory (*fiat*). The desired final inventory in the factory is calculated by the maximum of inventory coverage (*ic*) and the desired shipment rate (*dsr*) multiplied by the order processing time (*opt*). We use the maximum here to make sure that high order backlogs which arise when factory inventory levels are too low, will eventually be depleted even when current order rates coming from Norway are lower than the backlog. The inventory coverage (*ic*) is equal to the order rate (*or*) times the average shipment time (*ast*).

$RMF\left( t \right)=RMF\left( 0 \right)+\int_{0}^{t} \left( pur\left( s \right)-pr(s) \right)ds;RMF\left( 0 \right)=dRM(0)$ tests

$pur\left( t \right)=MAX(0,dprRM(t))$ tests/day

$dprRM\left( t \right)=PF\left( t \right)+(dRM(t)-RMF(t))/fiat$ tests/day

$dRM\left( t \right)=PF\left( t \right)*apt$ tests

$PF\left( t \right)=PF\left( 0 \right)+\int_{0}^{t} \left( cPF\left( s \right) \right)ds;PF\left( 0 \right)=dpr(0)$ tests/day

$cPF\left( t \right)=(dpr\left( t \right)-PF(t))/ffat$ tests/day/day

Raw materials for diagnostic tests (*RMF*) required to produce diagnostic tests in the factory are purchased from an external supplier (not included in our model). This purchasing rate (*pur*) follows the desired purchasing rate of raw materials (*dprRM*), which in turn is determined by a production forecasts (*PF*) and a raw material inventory adjustment. The latter is calculated by the difference between the desired raw material inventory (*dRM*) minus the raw material in factory (*RMF*) divided by the factory inventory adjustment time (*fiat*). The desired raw material inventory (*dRM*) is equal to the multiplication of the production forecast (*PF*) and the average production time (*apt*). Finally, the production forecast (*PF*) is based on the desired production rate (*dpr*), and a factory forecast adjustment time (*ffat*). We have assumed that there are no limits or constraints when it comes to purchasing raw materials.

$tur\left( t \right)=MIN(FI(t)/tut,ttr(t)*tpp/ttp)$ tests/day

$ttr\left( t \right)=trbs\left( t \right)+arhq(t)$ tests/day

$ptr\left( t \right)=((FI\left( t \right)*ttp)/tpp)/att$ people/day

$ptrbs\left( t \right)=MAX(0,ptr(t)-arhq(t))$ people/day

Finally, we must link this diagnostic test supply chain subsystem to the COVID-19 transmission subsystem, as the inventory of diagnostic tests will determine how many people can get tested. Firstly, the diagnostic test usage rate (*tur*) is determined by final inventory level (*FI*), the total test rate (*ttr*), which is the sum of the test rate before symptoms (*trbs*) and the admission rate to hospital from quarantine (*arhq*). These two variables have been explained in Appendix 1. The maximum test rate that is possible (*ptr*) is constrained by the available diagnostic test inventory (*FI*). When inventory is short, we have assumed that priority is given to testing people who are in quarantine but are so sick that they need to be hospitalized (*arhq*). If there are diagnostic tests remaining, these diagnostic tests are used to test people that suspect that they are infected, even though they have not experienced symptoms yet (*trbs*).

**Appendix 3. Model Documentation of the Policy Interventions Subsystem**

Figures E and F below show the stocks, flows and variables used to model the policy interventions that were used in Norway to flatten the curve. Values for the blue underlined bold exogenous variables were chosen based on the dates of the real major policy interventions that were implemented by the Norwegian government (for a timeline of these interventions, see <https://www.vg.no/spesial/corona/tiltak/>). Values for the blue variables were found through calibrating the model with real data about COVID-19 infections in Norway over time (this will be described in Appendix 4). Green variables link this subsystem to the COVID-19 transmission subsystem (described in Appendix 1). When simulating what-if scenarios the curves of infections may be changed (flatter or steeper, starting earlier or later), which is why we made policy interventions endogenous. This means interventions depend on either the cumulative cases of COVID-19 (*CC*) or the new infections per day (*ttr*). When a certain threshold is reached, interventions start or end. The way we modeled the start and end days of interventions is depicted in Figure E. Most interventions were targeted at reducing the number of contacts between people and reducing the possibility to be infected by focusing on hygienic factors like washing hands, face masks, etc. We have aggregated these interventions in the variables “Lockdown Factor (*L*)” and “Hygiene Focus Factor (*H*)”. The way these variables are modeled is depicted in Figure F.

**Figure E. Stocks, flows and variables of the policy interventions subsystem: start and end days of interventions**

**Figure F. Stocks, flows and variables of the policy interventions subsystem: lockdown factor and hygiene focus factor**

**Table D. Policy Interventions Subsystem**

**Formulations and comments Units**

$DSH1\left( t \right)=DSH1\left( 0 \right)+\int_{0}^{t} \left( cDSH1\left( s \right) \right)ds;DSH1\left( 0 \right)=0$ dmnl

$cDSH1\left( t \right)=IF THEN ELSE(CC(t)\geq tsH1,scp,0)$ dmnl/day

$CC\left( t \right)=CC\left( 0 \right)+\int_{0}^{t} \left( ttr\left( s \right) \right)ds;CC\left( 0 \right)=0$ people

$sH1\left( t \right)=IF THEN ELSE(DSH1\left( t \right)>0, 1,0)$ dmnl

$eH1\left( t \right)=DELAY FIXED\left( sH1\left( t \right),lH1,0 \right)$ dmnl

All six stocks shown in Figure E behave in a similar way. These stocks have the value 0 until some threshold is reached, after which the value of these stocks starts to increase. The exact value of these stocks is irrelevant, only the time of the first increase, from the value 0 is important, as this indicates the time at which a certain policy intervention starts or ends. The day to start with an increased hygiene focus in the first wave (*DSH1*) depends on the number of cumulative cases (*CC*). When this value exceeds the threshold for start of hygiene focus 1 (*tsH1*), the stock will start increasing with a constant value determined by a so-called signal to change policy (*scp*). Because this threshold is the same for all scenarios, a scenario that leads to a faster (slower) increase of cumulative cases will have an earlier (later) day to increased hygiene focus. Finally, the day to start hygiene focus 1 (*DSH1*) is used to calculate the start day of increased hygiene focus 1 (*sH1*). This variable has the value 0 and jumps to 1 when the focus on hygiene is increased. The duration (length) of the hygiene focus in wave 1 (*lH1*) determines the end day of increased hygiene focus 1 (*eH1*). The thresholds that we have used to start and end policy interventions are calibrated with the real start and end dates of policy interventions in Norway. For example, Norway started to promote an increased focus on washing hands on day 31. On this day the (simulated) cumulative number of infected people was 55.48. Therefore, we set the threshold for starting a focus on hygiene (*tsH1*) on 55.48.

$DSH2\left( t \right)=DSH2\left( 0 \right)+\int_{0}^{t} \left( cDSH2\left( s \right) \right)ds;DSH2\left( 0 \right)=0$ dmnl

$cDSH2\left( t \right)=IF THEN ELSE(time>e1:AND: ttr(t)\geq tsH2,scp,0)$ dmnl/day

$sH2\left( t \right)=IF THEN ELSE(DSH2\left( t \right)>0,1,0)$ dmnl

$eH2\left( t \right)=DELAY FIXED(sH2(t),lH2,0)$ dmnl

To calculate the start day of increased hygiene focus in the second wave, we did not use the cumulative number of cases. This is because different scenarios lead to huge differences in cumulative cases, such that policy interventions would start unrealistically late or not at all in the second wave. Therefore, we use the daily new infections (*ttr*). When the *ttr* exceeds the threshold (*tsH2*) after the first wave is over (*e1*), the second wave of increased hygiene focus begins. It ends (*eH2*) after the length of the second hygiene focus wave (*lH2*).

$bH1\left( t \right)=IF THEN ELSE(sH1\left( t \right)=0,1,0)$ dmnl

$dH1\left( t \right)=sH1(t)-eH1(t)$ dmnl

$$bH1H2\left( t \right)=$$

$IF THEN ELSE(bH1(t)=0:AND:dH1(t)=0:AND:dH2(t)=0:AND:bH2H3(t)=0,1,0)$ dmnl

$dH2\left( t \right)=sH2\left( t \right)-eH2(t)$ dmnl

$bH2H3\left( t \right)=IF THEN ELSE(time>e2:AND:dH2(t)=0,1,0)$ dmnl

With the start and end days of the first and second wave of increased hygiene focus, we can define 5 different periods: the period before the first hygiene focus wave (*bH1*), the period during the first hygiene focus wave (*dH1*), the period between the first and second hygiene focus wave (*bH1H2*), the period during the second hygiene focus wave (*dH2*) and the period after the second hygiene focus wave (potentially a period between the second and third hygiene focus wave, *bH2H3*). These periods will be used to determine the hygiene focus factor (*H*) of people over time.

$H\left( t \right)=H\left( 0 \right)+\int_{0}^{t} \left( cH\left( s \right) \right)ds;H\left( 0 \right)=iH$ dmnl

$cH\left( t \right)=dH1(t)*\frac{iH1-H(t)}{dtms}+bH1H2(t)*\frac{iH-H(t)}{dchf}+dH2(t)*\frac{0}{dtms}+bH2H3(t)*\frac{iH-H(t)}{dchf}$ dmnl/day

$effi\left( t \right)=i*H(t)$ dmnl

The hygiene focus factor (*H*) changes from period to period. In normal situations, this factor is 1, but it can decrease when policy interventions are deployed. Changing *H* is modeled as a first-order material delay. Decreasing the hygiene focus factor, which corresponds to more hand washing, etc., occurs faster (delay in taking measures seriously, *dtms*) than increasing the hygiene focus factor (delay in changing hygiene focus, *dchf*). Initially, the hygiene focus factor is equal to 1 (*iH*). During the first and second wave, this factor adjusts to new values. In the first wave, the new factor is defined by the infectivity due to hygiene focus 1 (*iH1*), a value found through calibration (see Appendix 4). In the second wave (*dH2*) we have assumed focus on hygiene does not change but remains at the same level as before the start of the second wave. The hygiene focus factor has a value that is always between 0 and 1. The multiplication of *H* with the normal infectivity (*i*) of COVID-19 will lead to the effective infectivity of COVID-19 (*effi*). This *effi* is used in the COVID-19 transmission subsystem (see Appendix 1).

$DSL1\left( t \right)=DSL1\left( 0 \right)+\int_{0}^{t} \left( cDSL1\left( s \right) \right)ds;DSL1\left( 0 \right)=0$ dmnl

$cDSL1\left( t \right)=IF THEN ELSE(CC(t)\geq tsL1,scp,0)$ dmnl/day

$sL1\left( t \right)=IF THEN ELSE(DSL1\left( t \right)>0,1,0)$ dmnl

$DSL2\left( t \right)=DSL2\left( 0 \right)+\int_{0}^{t} \left( cDSL2\left( s \right) \right)ds;DSL2\left( 0 \right)=0$ dmnl

$cDSL2\left( t \right)=IF THEN ELSE(time>e1 :AND:ttr(t)\geq tsL2,scp,0)$ dmnl/day

$sL2\left( t \right)=IF THEN ELSE(DSL2\left( t \right)>0,1,0)$ dmnl

The start days of the lockdowns in the first (*sL1*) and second (*sL2*) wave are defined in similar ways as *sH1* and *sH2*, so we only list the equations here without any explanations.

$DEL1\left( t \right)=DEL1\left( 0 \right)+\int_{0}^{t} \left( cDEL1\left( s \right) \right)ds;DEL1\left( 0 \right)=0$ dmnl

$cDEL1\left( t \right)=IF THEN ELSE(eeL1(t)=0,0, IF THEN ELSE\left( teL1\geq ttr\left( t \right),scp,0) \right)$ dmnl/day

$eeL1\left( t \right)=DELAY FIXED(sl1(t),mlL1,0)$ dmnl

$eL1\left( t \right)=IF THEN ELSE(DEL1\left( t \right)>0,1,0)$ dmnl

$DEL2\left( t \right)=DEL2\left( 0 \right)+\int_{0}^{t} \left( cDEL2\left( s \right) \right)ds;DEL2\left( 0 \right)=0$ dmnl

$cDEL2\left( t \right)=IF THEN ELSE(eeL2(t)=0,0, IF THEN ELSE\left( teL2\geq ttr\left( t \right),scp,0) \right)$ dmnl/day

$eeL2\left( t \right)=DELAY FIXED(sl2(t),mlL2,0)$ dmnl

$eL2\left( t \right)=IF THEN ELSE(DEL2\left( t \right)>0,1,0)$ dmnl

To calculate the end of the first lockdown 1 (*DEL1* and *eL1*), the daily new infections (*ttr*) need to reach a threshold (*teL1*). Furthermore, we check that the lockdown has a certain minimum duration (*mlL1*). Therefore, the shortest possible end day of lockdown 1 (*seL2*) depends on the start day of the lockdown (*sl2*) and this minimum duration or length of lockdown 1 (*mlL1*). Similar equations are used to calculate the end day of the second lockdown (*eL2*).

$bL1\left( t \right)=IF THEN ELSE(sL1\left( t \right)=0,1,0)$ dmnl

$dL1\left( t \right)=sL1\left( t \right)-eL1(t)$ dmnl

$$bL1L2\left( t \right)=$$

$IF THEN ELSE(bL1(t)=0:AND:dL1(t)=0:AND:dL2(t)=0:AND:bL2L3(t)=0,1,0)$ dmnl

$dL2\left( t \right)=sL2\left( t \right)-eL2(t)$ dmnl

$bL2L3\left( t \right)=IF THEN ELSE(time>e2:AND:dL2(t)=0,1,0)$ dmnl

With the start and end days of the first and second waves of locking down society, we can define 5 different periods: the period before the first lockdown (*bL1*), the period during the first lockdown (*dL1*), the period between the first and second lockdown (*bL1L2*), the period during the second lockdown (*dL2*) and the period after the second lockdown (potentially a period between the second and third lockdown, *bL2L3*). These periods will be used to determine the lockdown (*L*) of society over time.

$L\left( t \right)=L\left( 0 \right)+\int_{0}^{t} \left( cL\left( s \right) \right)ds;L\left( 0 \right)=iL$ dmnl

$cL\left( t \right)=dL1(t)*\frac{exL1-L(t)}{dtms}+bL1L2(t)*\frac{iL-L(t)}{dou}+dL2(t)*\frac{exL2-L(t)}{dtms}+bL2L3(t)*\frac{iL-L(t)}{dou}$ dmnl/day

$exL2=exL1*sevL2$ dmnl

$effc\left( t \right)=c*L(t)$ dmnl/day

$ci\left( t \right)=effc\left( t \right)*effi(t)$ dmnl/day

The lockdown factor (*L*) changes from period to period. These changes are modeled as first-order material delays. Increasing the lockdown occurs faster (delay in taking measures seriously, *dtms*) than decreasing the lockdown (delay in opening up, *dou*). Initially, the lockdown factor is equal to 1 (*iL*). During the first and second wave, this factor adjusts to new values that are given by the extent of lockdown 1 (*exL1*) and 2 (*exL2*). Note that while calibrating the model (explained in Appendix 4), we made sure the extent of the second lockdown factor (*eL2*) to be higher than the first, by introducing the severity of lockdown 2 (*sevL2*) which corresponds to what really happened in Norway (the second lockdown being less severe than the first one). These calculations lead to a lockdown factor that is always between 0 and 1. The multiplication of *L* with the normal contact rate (*c*) of COVID-19 will lead to the effective contact rate of COVID-19 (*effc*). This *effc* is used in the COVID-19 transmission subsystem. The effective contact rate multiplied by the effective infectivity (*ci*) is used to calculate the transmission (see Appendix 1). The perceived value of *ci* (*Pci*) is used to calculate expectations about the transmission (see Appendix 2.1).

**Appendix 4. Model Calibration**

**4.1. Calibration**

The model with all three subsystems that were described previously contains many exogenous variables (constants). The values of some of these variables were found in reports published by the Norwegian Institute of Public Health (<https://www.fhi.no/sv/smittsomme-sykdommer/corona/koronavirus-modellering/>). These variables are underlined and written in bold and blue font in Figures A, C, D, E, and F. Estimates of the values for the other blue variables in these figures were derived using time series and the least squares method (calibration). To estimate these variables, we used actual data about the number of new cases of COVID-19 in Norway per day, the cumulative number of cases, the COVID-19 patients in hospitals, in intensive care units, and the cumulative number of deceased patients. For the first two data series we used the website of the Norwegian Institute of Public Health (<https://www.fhi.no/sv/smittsomme-sykdommer/corona/koronavirus-modellering/>). This site was also used to find data on the number of diagnostic tests used (but these data were not used for calibration purposes). For the last three data series we used the Norwegian newspaper VG (<https://www.vg.no/spesial/corona/>). Calibration was executed in two steps. First, we calibrated the “front-end” of the model: making sure that the simulated number of daily cases (*ttr*) and cumulative cases (*CC*) matches the real numbers. Then, while using the values found during the calibration of the “frond-end”, we calibrated the “back-end” of the model: making sure the simulated number of people in hospitals (*IH*), ICUs (*IIC*), and dying from COVID-19 (*DP*) matches the real numbers. The calibration problems for the front-end and back-end are stated in Table E and F. The estimates we derived for these blue variables, their confidence intervals, and the summary statistics are shown in Table G.

The Theil inequality statistics presented in Table G describe the fraction of the mean square error between simulated and actual series that is due to unequal means (bias), unequal variances, and imperfect correlation (Oliva, 2003). Low bias and variance fractions indicate that the error is unsystematic (Sterman, 1984). In this case, the model can endogenously generate the behavior that marks the observed system (Sterman, 1984). As Table G shows, the fit to historical behavior (real data) is quite good. The largest part of the MSE is concentrated in the imperfect covariation component of the Theil inequality statistics. For the five data series included in the calibration, the covariation was respectively: 0.9646, 0.9984, 0.8126, 0.9768, and 0.9375. R^2^ is very high. For the five data series included in the calibration process we found respectively 0.8480, 0.9977, 0.9389, 0.9127, and 0.9890.

The estimated parameters all have the expected sign and tight confidence intervals. Because of this high fit, we are confident that our model is a good reflection of the real situation in Norway and can be used for further analysis, like what-if simulations. As we explained in Appendix 2.1, policymakers do not know the pre-symptomatic infection rate (*pir*), but they can calculate the expected pre-symptomatic infection rate (*epir*). To calculate the epir, policymakers use a SEIR-model. Some variables in the SEIR-model were unknown to us, so we included them in the calibration process: the expected initial infectious pre-symptomatic population (*eiIPP*) and the expected relative infectivity of infectious population in quarantine (*eriIQ*). The calibrated value for *eiIPP* is 0.292285. The calibrated value for the “real” initial infectious pre-symptomatic population (*initial IPP*) is 0.313049. This suggests that policymakers underestimated the start of the pandemic somewhat (as their expectancies start with a lower value). The calibrated value for *eriIQ* is 0.39917. The calibrated value for the “real” relative infectivity of the infectious population in quarantine (*riIQ*) is 0.473248. This suggests that policymakers also underestimate the willingness of people to go in quarantine when they suspect that they may be infected. Although we do not know if the size of these underestimations is correct, the fact that policymakers underestimated the infection rates is behavior that is recognizable and also led to the diagnostic test shortages during the contagion phase.

As the Norwegian Institute of Public Health does not publish real values of the reproduction rate (*R*) frequently, we did not include *R* in our calibration process. We did perform a visual inspection of the real data on *R* we were able to find with an estimate of *R* (*Re*) based on our simulation results (<https://www.fhi.no/contentassets/8a971e7b0a3c4a06bdbf381ab52e6157/vedlegg/andre-halvar-2020/2021.01.06-ukerapport-uke-53-covid-19.pdf>). Our simulated *R* (*Re*) depends on the contact rate, infectivity rate and an average time used to calculate R (*atR*). As such, we calculated this reproduction estimate (*Re*) as follows:

$Re\left( t \right)=effc\left( t \right)*effi\left( t \right)*atR$ dmnl

Figure G shows our simulated *Re* and the real data we were able to find on *R* in Norway.


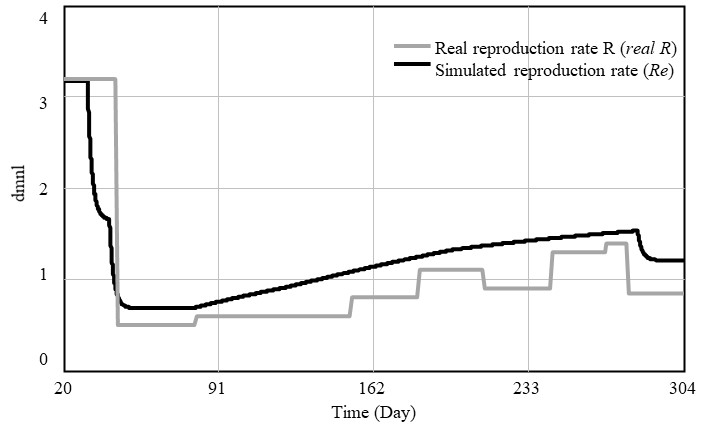


**Figure G. Simulated (*Re*) versus reported values of reproduction rate (*real R*)**

**Table E. Calibration problem for the “frond-end”**

**Minimize for 1 ≤ t ≤ 304 (from February 1, 2020 until December 1, 2020):**

$\sum\left( ttr\left( t \right)-real ttr\left( t \right) \right)^{2}$

$\sum\left( CC\left( t \right)-real CC\left( t \right) \right)^{2}$

$\sum\left( FI\left( t \right)*ttp-real ttr\left( t \right) \right)^{2}$

$\sum\left( epir\left( t \right)-pir\left( t \right) \right)^{2}$

**Over:**

2.5 ≤ *c* (normal contact rate) ≤ 6

28 ≤ *dchf* (delay in changing hygiene focus) ≤ 750

14 ≤ *dou* (delay in opening up) ≤ 250

0.1 ≤ *eiIPP* (expected initial infectious pre-symptomatic population) ≤ 0.5

0.2 ≤ *eriIQ* (expected relative infectivity of infectious population in quarantine) ≤ 0.9

0.25 ≤ *exL1* (extent of lockdown 1) ≤ 0.75

1 ≤ *fat* (forecast adjustment time) ≤ 14

0.15 ≤ *i* (normal infectivity) ≤ 0.5

0.25 ≤ *iH1* (infectivity due to hygiene focus 1) ≤ 0.75

0.001 ≤ *initial IPP* (initial infectious pre-symptomatic population) ≤ 0.5

3000000 ≤ *initial N* (initial total population) ≤ 5000000

2000000 ≤ *initial S* (initial susceptible population) ≤ 4000000

0.1 ≤ *mic* (min inventory coverage) ≤ 4

0.25 ≤ *riIQ* (relative infectivity of infectious population in quarantine) ≤ 1

1 ≤ *sevL2* (severity of lockdown 2) ≤ 1.7

**Subject to:**

All equations described in the previous appendices.

**Table F. Calibration problem for the “back-end”**

**Minimize for 1 ≤ t ≤ 304 (from February 1, 2020 until December 1, 2020):**

$\sum\left( IH\left( t \right)-real IH\left( t \right) \right)^{2}$

$\sum\left( IIC\left( t \right)-real IIC\left( t \right) \right)^{2}$

$\sum\left( DP\left( t \right)-real DP\left( t \right) \right)^{2}$

**Over:**

0 ≤ *mph1* (multiplier percentage to hospital 1) ≤ 1

0 ≤ *mph2* (multiplier percentage to hospital 2) ≤ 2

0 ≤ *mpic1* (multiplier percentage to ICU 1) ≤ 6

0 ≤ *mpic2* (multiplier percentage to ICU 2) ≤ 14

0.1 ≤ *ndp* (normal death percentage) ≤ 1

**Subject to:**

All equations described in the previous appendices.

**Table G. Calibration results**

| **Simulated versus Real data** | **Summary Statistics** |
| --- | --- |
| **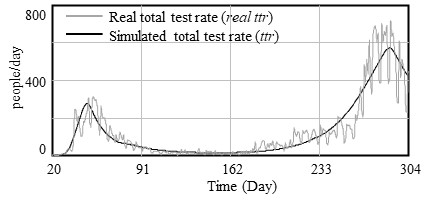** | n = 278  R2 = 0.847974037  MAPE = 0.813238  RMSE =66.092095  **Theil’s inequality statistics:**  Bias = 0.000728  Unequal variation = 0.034661  Unequal covariation = 0.964611 |
| **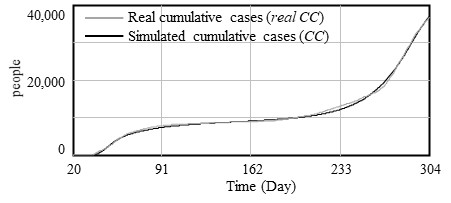** | n = 284  R2 = 0.997700421  MAPE = 0.116487  RMSE = 373.508770  **Theil’s inequality statistics:**  Bias = 0.000000  Unequal variation = 0.001564  Unequal covariation = 0.998436 |
| **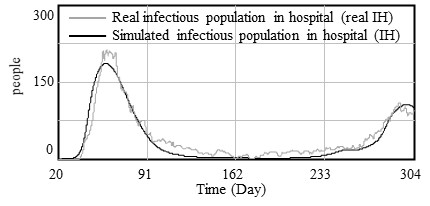** | n = 267  R2 = 0.938912992  MAPE = 0.471230  RMSE = 14.357100  **Theil’s inequality statistics:**  Bias = 0.149438  Unequal variation = 0.037986  Unequal covariation = 0.812576 |
| **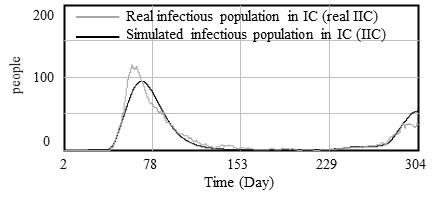** | n = 252  R2 = 0.912667153  MAPE = 0.335667  RMSE = 8.296077  **Theil’s inequality statistics:**  Bias = 0.011936  Unequal variation = 0.011264  Unequal covariation = 0.976800 |
| **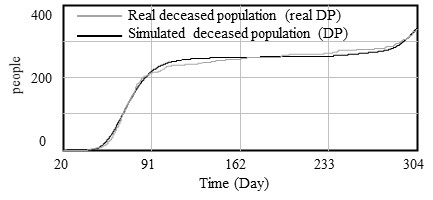** | n = 264  R2 = 0.988979131  MAPE = 0.062908  RMSE = 8.304068  **Theil’s inequality statistics:**  Bias = 0.004101  Unequal variation = 0.058424  Unequal covariation = 0.937475 |
| **Confidence Intervals**  **Estimate used in front-end**  *c,* normal contact rate = 3.09763  *dchf,* delay in changing hygiene focus = 598.431  *dou,* delay in opening up = 158.778  *eiIPP*, expected initial infectious pre-sympt. pop. = 0.292285  *eriIQ*, expected relative infectivity of IQ = 0.39917  *exL1,* extent of lockdown 1 = 0.412605  *fat,* forecast adjustment time = 5.38964  *i,* normal infectivity = 0.455379  *iH1,* infectivity due to hygiene focus 1 = 0.51788  *initial IPP*, initial infectious pre-sympt. pop. = 0.313049  *initial N,* initial total population = 4947880  *initial S*, initial susceptible population = 2009110  *mic,* min inventory coverage = 0.157191  *riIQ,* relative infectivity of IQ = 0.473248  *sevL2,* severity of lockdown 2 = 1.62589  **Estimate used in back-end**  *mph1,* multiplier percentage to hospital 1 = 0.151231  *mph2,* multiplier percentage to hospital 2 = 0.0906175  *mpic1*, multiplier percentage to ICU 1 = 2.68311  *mpic2*, multiplier percentage to ICU 2 = 8.08707  *ndp,* normal death percentage = 0.849583 | **95% confidence interval**  3.09602 - 3.09923  590.863 - 606.244  158.240 - 159.319  0.288339 - 0.296197  0.398508 - 0.39984  0.411697 - 0.413502  4.92832 - 5.85859  0.455143 - 0.455614  0.517398 - 0.518364  0.294998 - 0.347208  4945330 - 4950450  2008120 - 2010100  0.15625 - 0.158236  0.471251 - 0.475522  1.46227 - 1.70000  **95% confidence interval**  0.148681 - 0.153779  0.0802119 - 0.100999  2.62882 - 2.73768  6.23268 - 10.0348  0.835107 - 0.864043 |

**4.2. Comparison of real and simulated inventory gap of diagnostic tests**

We did not have access to real data about ordering, producing, and shipping diagnostic tests to Norway. Therefore, we chose to model these processes by using a standard stock management structure used by Sterman (2000). However, we do know how many people got tested and how many diagnostic tests were used. This information we used to find the value for the target test percentage (*ttp*), described earlier. A key variable in our model is the inventory gap (*ig*). When simulating the model, the inventory gap oscillates between positive gaps (shortage of diagnostic tests) and negative gaps (surplus of diagnostic tests). We checked public reports (newspapers, sites of health institutions) to validate whether our simulated periods of shortage and surplus overlap with what these reports write about the diagnostic test inventory situation in Norway. Figure H provides an overview of the simulated inventory gap (we normalized the values to make them fit between -1 and 1). The periods of shortage and surplus, according to our simulation results, are marked with vertical dotted lines. The dots in Figure H indicate a public report that writes about a shortage (value 1) or about a surplus (value -1). These public reports are listed in Table H.


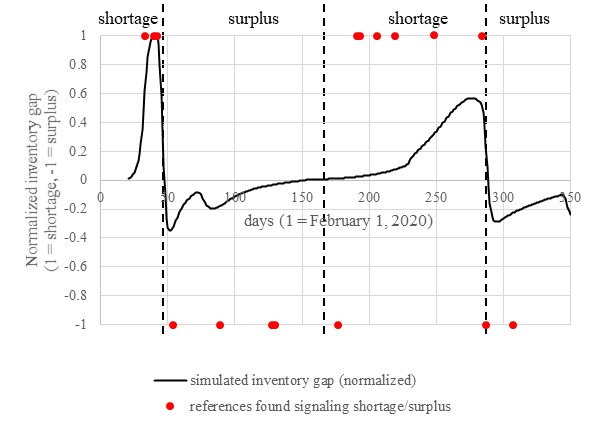


**Figure H. Simulated inventory gap compared to statements about the real inventory gap**

**Table H. List of public reports about diagnostic test shortages or surpluses**

| **Date published** | **Day in simulation** | **Shortage (1) or**  **surplus (-1)** | **Source** |
| --- | --- | --- | --- |
| 4-Mar-20 | 33 | 1 | https://www.aftenposten.no/norge/i/6j7z8r/presset-testkapasitet-  bemanning-og-mangel-paa-utstyr-vi-kan-faa-bety |
| 11-Mar-20 | 40 | 1 | https://www.abcnyheter.no/helse-og-livsstil/helse/2020/03/11/195655607/  derfor-blir-ikke-flere-testet-for-coronavirus |
| 13-Mar-20 | 42 | 1 | https://sykehuset-innlandet.no/fag-og-forskning/tjenester/  laboratorietjenester/koronavirus-og-testing |
| 18-Mar-20 | 54* | -1 | https://www.nrk.no/norge/langt-flere-kommer-til-a-bli-korona-testet-  fremover-1.14949807 |
| 29-Apr-20 | 89 | -1 | https://www.klartale.no/norge/alle-med-symptomer-pa-korona-kan-  bli-testet-1.1707768 |
| 7-Jun-20 | 128 | -1 | https://www.nrk.no/norge/helsesykepleiere-ma-prioritere-korona-arbeid-  fremfor-barn-og-unge-1.15041866 |
| 9-Jun-20 | 130 | -1 | https://www.nrk.no/norge/lag-pagang-for-a-teste-seg-for-korona-1.15045112 |
| 26-Jul-20 | 177 | -1 | https://forskning.no/epidemier-medisinske-metoder-virus/storstilt-dugnad-  sikret-norge-nok-korona-testerda-universitetet-matte-starte-fabrikk/1716367 |
| 9-Aug-20 | 191 | 1 | https://www.vg.no/nyheter/meninger/i/b5yVGe/vi-vil-ikke-tilbake-til-mars-  igjen |
| 11-Aug-20 | 193 | 1 | https://www.nrk.no/norge/norges-tre-storste-byer-klarer-ikke-a-oppfylle-  testkravene-1.15118416 |
| 24-Aug-20 | 206 | 1 | https://www.nrk.no/norge/okt-testkapasitet-gar-ut-over-andre-helsetjenester_-_-vi-taler-ikke-at-noen-blir-syke-1.15128873 |
| 6-Sep-20 | 219 | 1 | https://www.aftenposten.no/norge/i/JoKejR/norge-tester-seg-som-aldri-foer-  i-jakten-paa-viruset-naa-advarer-fagfolk |
| 6-Oct-20 | 249 | 1 | https://www.nrk.no/norge/hoie_-har-sikret-norge-5-millioner-hurtigtester-  1.15188713 |
| 10-Nov-20 | 284 | 1 | https://www.h-a.no/2020/nyheter/nei-du-trenger-ikke-negativ-korona-test-  for-a-komme-ut-av-isolasjon/ |
| 13-Nov-20 | 287 | -1 | https://www.helsedirektoratet.no/tema/beredskap-og-krisehandtering/  koronavirus/anbefalinger-og-beslutninger/Til%20landets%20kommuner%  20regionale%20helseforetak%20og%20fylkesmenn%20-%20Orientering%  20om%20evaluering%20av%20antigen%20hurtigtest%20og%20bruk%20  under%20utbrudd.pdf/_/attachment/inline/881a7977-d2d6-4941-b4b5-49c  15a2a495f:240c5b3f4aeb8f341278a30e4f42c7dd5d1fa18f/Til%20  landets%20kommuner%20regionale%20helseforetak%20og%20fylkesmenn  %20-%20Orientering%20om%20evaluering%20av%20antigen%20  hurtigtest%20og%20bruk%20under%20utbrudd.pdf |
| 3-Dec-20 | 307 | -1 | https://www.tv2.no/a/11813667/ |

* this report was published on day 47, but as the article writes about an improved situation in a week, we marked this statement on day 54

Because of the lack of real data on the inventory gap, we cannot perform any statistical analyses on model fit. We can only use visual inspection of Figure H. The simulated periods of diagnostic test inventory shortage and surplus and the identified public reports about shortage and surplus have a good overlap. The model seems to reproduce behavior of interest (oscillation of inventory gaps) and it does this endogenously (not because we make a variable jump up and down). Therefore, we conclude that our model can be used for further what-if simulations.

**Appendix 5. Robustness and Sensitivity**

**5.1 Robustness checks**

We have performed four types of robustness checks with our model. First, we checked the results of our model with different time steps. Second, we changed the integration methods to evaluate if these methods influence our results. Third, we have checked whether our model can handle a large increase in the percentage of people that needs to be hospitalized. Fourth and finally, we have checked to see if the model is able to handle an introduction of a mutant virus and if so, how this impacts the results.

**5.1.1 Changing time steps**

Our model uses the 0.0625 days as a time step, which that every 1.5-hour calculations are made. We believe this time step is accurate, as in real life, changes could also be noticeable within 1.5 hour. A person that becomes so sick that hospitalization is required will not have to wait for a day (at least not in Norway). Or when admission to the ICU is required, this will also happen almost immediately. Nevertheless, it makes sense to check our results and recommendations when we select even shorter or longer time steps. We have simulated our base case (without collaborating with other countries) and our best case (accepting and donating 100% of inventory shortages and surpluses) for five different time steps (0.0625 is the reference value used for the simulations we discussed in the manuscript). Table I and Figures I and J present the results. In general, we see for longer time steps the results deteriorate (due to the somewhat longer delays). Nevertheless, the best case is still much better than the base case, for all time steps used. Also, as Figures I and J show, the behavior over time is similar for all time steps. Therefore, we conclude that our model is robust for changes in the time step.

**Table I. Comparison of scenarios with different time steps**

| **Scenario** | **Days in Lockdown Wave 1** | **Total COVID-19 Cases** | **Max. Infectious Population in Hospital Wave 1** | **Total deaths** | **Total Tests Shipped to Norway** |
| --- | --- | --- | --- | --- | --- |
| base case time step 0015625 | 37.6 | 53685.2 | 185.1 | 496.5 | 3362660 |
| base case time step 003125 | 38.0 | 53728.3 | 186.8 | 498.3 | 3365370 |
| base case time step 00625 | 38.0 | 53740.1 | 187.5 | 499.0 | 3366110 |
| base case time step 0125 | 39.6 | 53987.2 | 195.4 | 507.0 | 3381610 |
| base case time step 025 | 41.0 | 54193.7 | 203.9 | 513.5 | 3394580 |
|  |  |  |  |  |  |
| +-100% of short. & surpl. time step 0.015625 | 25.0 | 51641.3 | 111.8 | 382.1 | 3234710 |
| +-100% of short. & surpl. time step 0.03125 | 25.1 | 51653.1 | 112.3 | 383.1 | 3235440 |
| +-100% of short. & surpl. time step 0.0625 | 25.2 | 51692.2 | 113.8 | 385.3 | 3237880 |
| +-100% of short. & surpl. time step 0.125 | 25.9 | 51797.6 | 118.3 | 393.6 | 3244470 |
| +-100% of short. & surpl. time step 0.25 | 26.3 | 51891.9 | 123.1 | 401.8 | 3250370 |

**
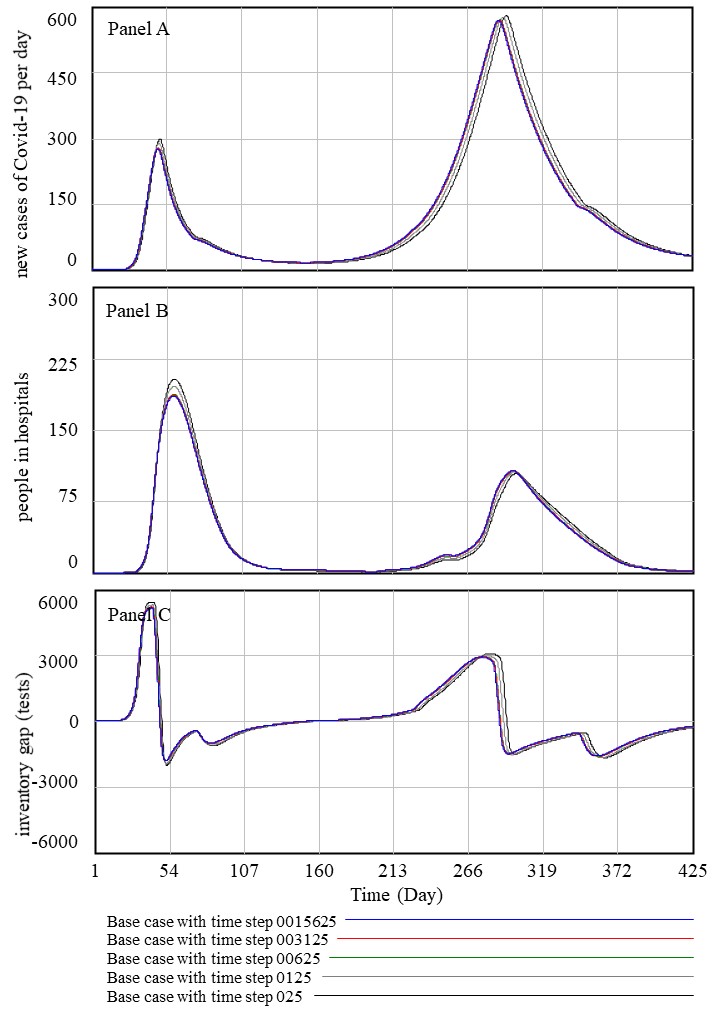
**

**Figure I. Base case simulation with different time steps (0.0625 is reference)**

**
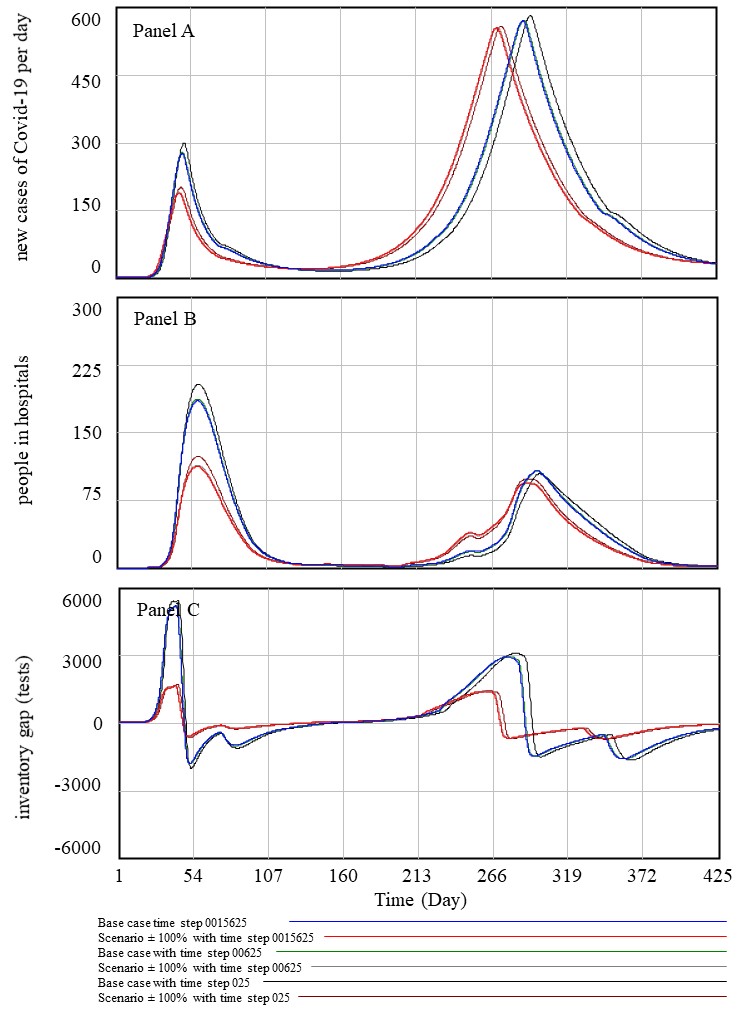
**

**Figure J. Simulation of base case and best case with three different time steps**

**5.1.2 Changing integration methods**

Our model uses Euler as the integration method. Euler integration is the simplest and most obvious way to numerically integrate a set of differential equations. Euler integration assumes that the rates computed at a given time are constant through the time interval (the time step). In general, this is not likely to be true, which could make simulation results less accurate. However, since we have chosen a small time step, we do not see a problem with using Euler integration. Nevertheless, we have also checked our results and recommendations when we select different integration methods. (For more information about these integration methods we refer to: <https://www.vensim.com/documentation/rungekutta.html>. We have simulated our base case (without collaborating with other countries) and our best case (accepting and donating 100% of inventory shortages and surpluses) for five different integration methods. Besides Euler, we have used RK2-auto, RK2-fixed, RK4-auto, and RK4-fixed. Table J and Figures K and L present the results. In general, the base case results and the best case results are very similar, regardless of the integration method used. Also, as Figures K and L show, the behavior over time is similar for all time steps. Therefore, we conclude that our model is robust for changes in integration method.

**Table J: Comparison of scenarios with different integration methods**

| **Scenario** | **Days in Lockdown Wave 1** | **Total COVID-19 Cases** | **Max. Infectious Population in Hospital Wave 1** | **Total deaths** | **Total Tests Shipped to Norway** |
| --- | --- | --- | --- | --- | --- |
| base case Euler | 38.0 | 53740.1 | 187.5 | 499.0 | 3366110 |
| base case RK2 auto | 37.3 | 53656.4 | 184.1 | 495.4 | 3360850 |
| base case RK2 fixed | 37.9 | 53719.1 | 186.0 | 497.5 | 3364790 |
| base case RK4 auto | 37.3 | 53646.9 | 183.9 | 495.1 | 3360260 |
| base case RK4 fixed | 37.6 | 53680.5 | 184.8 | 496.2 | 3362360 |
|  |  |  |  |  |  |
| +-100% of short. & surpl. Euler | 25.2 | 51692.2 | 113.5 | 385.3 | 3237880 |
| +-100% of short. & surpl. RK2 auto | 24.9 | 51629.3 | 111.2 | 381.1 | 3233960 |
| +-100% of short. & surpl. RK2 fixed | 25.1 | 51647.7 | 112.0 | 382.5 | 3235100 |
| +-100% of short. & surpl. RK4 auto | 24.9 | 51618.2 | 111.0 | 380.8 | 3233260 |
| +-100% of short. & surpl. RK4 fixed | 25.0 | 51641.1 | 111.6 | 381.9 | 3234690 |

**
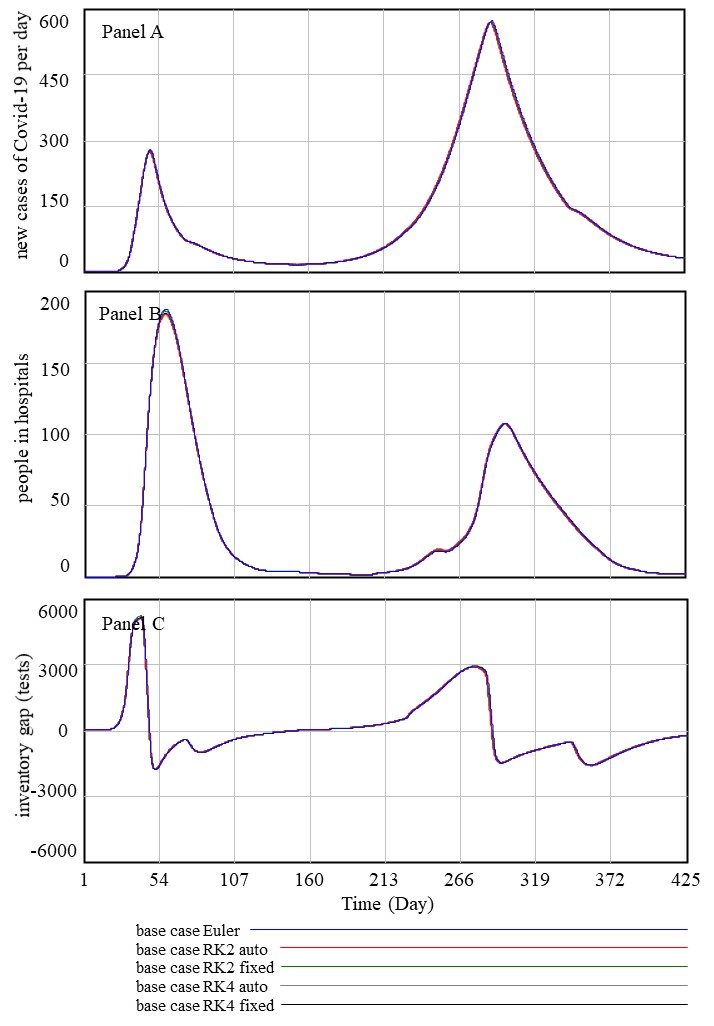
**

**Figure K. Base case simulation with different integration methods (Euler is reference)**

**
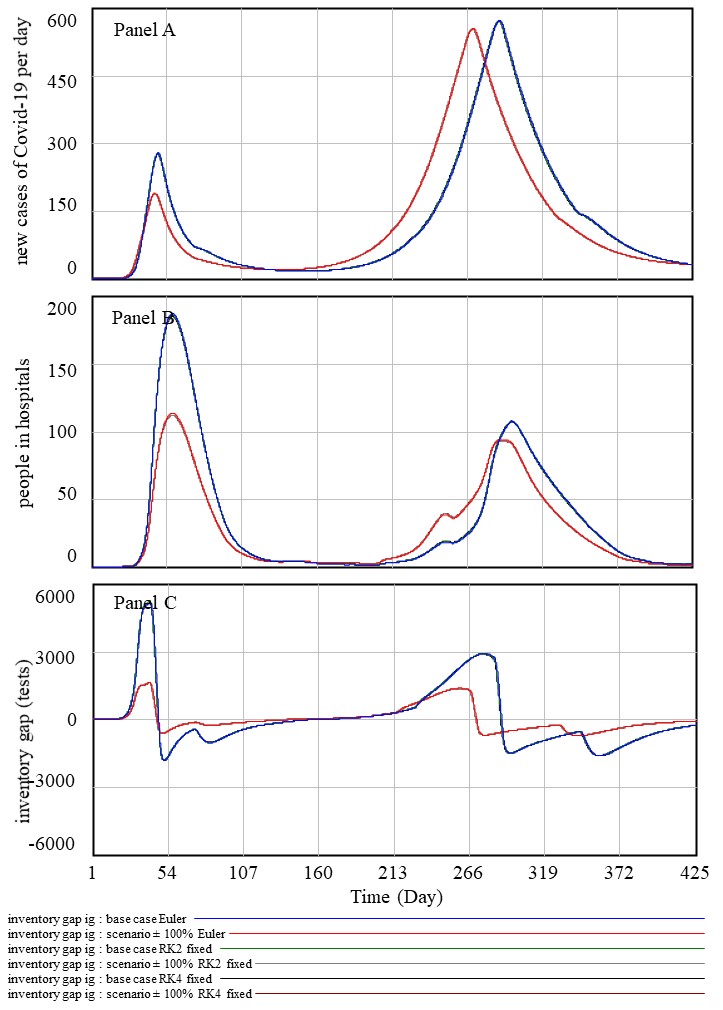
**

**Figure L. Simulation of base case and best case (scenario ± 100%) with three different integration methods (Euler is reference)**

**5.1.3 Increasing hospitalizations**

As we explained in Appendix 1, we assume that when someone in quarantine becomes so sick that hospitalization is required, this person will get tested before admission (*arhq*). This means that there should always be diagnostic tests available for this purpose, otherwise, we should have modeled a diagnostic test backlog for hospitals. The available inventory of diagnostic tests is first allocated to the people that need to be tested when they go from a voluntary quarantine to the hospital (*arhq*). The remaining number of tests can be allocated to people that would like to get tested because they suspect that they may be infected, but they have not discovered any symptoms yet (*trbs*). Low inventory levels therefore imply that this *trbs* will be close to 0, as the *arhq* will be prioritized. Using the percentage to hospital (*ph*) as described in Appendix 1 and 4, will not lead to any inventory shortage for the arhq, which implies that modeling a diagnostic test backlog in the hospital is not required. Nevertheless, we need to check if this is also true when *ph* is higher, leading to a higher arhq and a higher need for diagnostic tests. We have therefore simulated our base case with different a percentage to hospital ranging from twice as high as in our base case (ph x 2) to ten times as high (ph x 10). The results are presented in Figure M. Panel A in Figure M shows the different percentages to hospital we simulated. Panel B shows that these percentages hardly influence the cumulative number of people that get infected. Actually, more hospitalizations reduce this number somewhat, as we have assumed that people in hospitals do not infect other people anymore. Panel C confirms that for higher values of ph, the rate of people that need to be admitted to hospitals (*arhq*) also increases. Panel D shows for which levels of ph there are not sufficient diagnostic tests available anymore to test “healthy” people that only suspect they may be infected (*trbs*). Between around day 60 and 80 we see that for higher values of *ph* these number drop to 0, implying that all diagnostic tests are allocated to *arhq* which could lead to shortages of diagnostic tests in hospitals. This happens for values of *ph* which are six times higher than the values we used in the base case. This is such an extreme situation, that leads to a population in hospitals that is far from what has ever been seen in Norway. Therefore, we conclude that, although leaving out a backlog of diagnostic tests is a simplification, it does not impact our simulation results or recommendations.

**
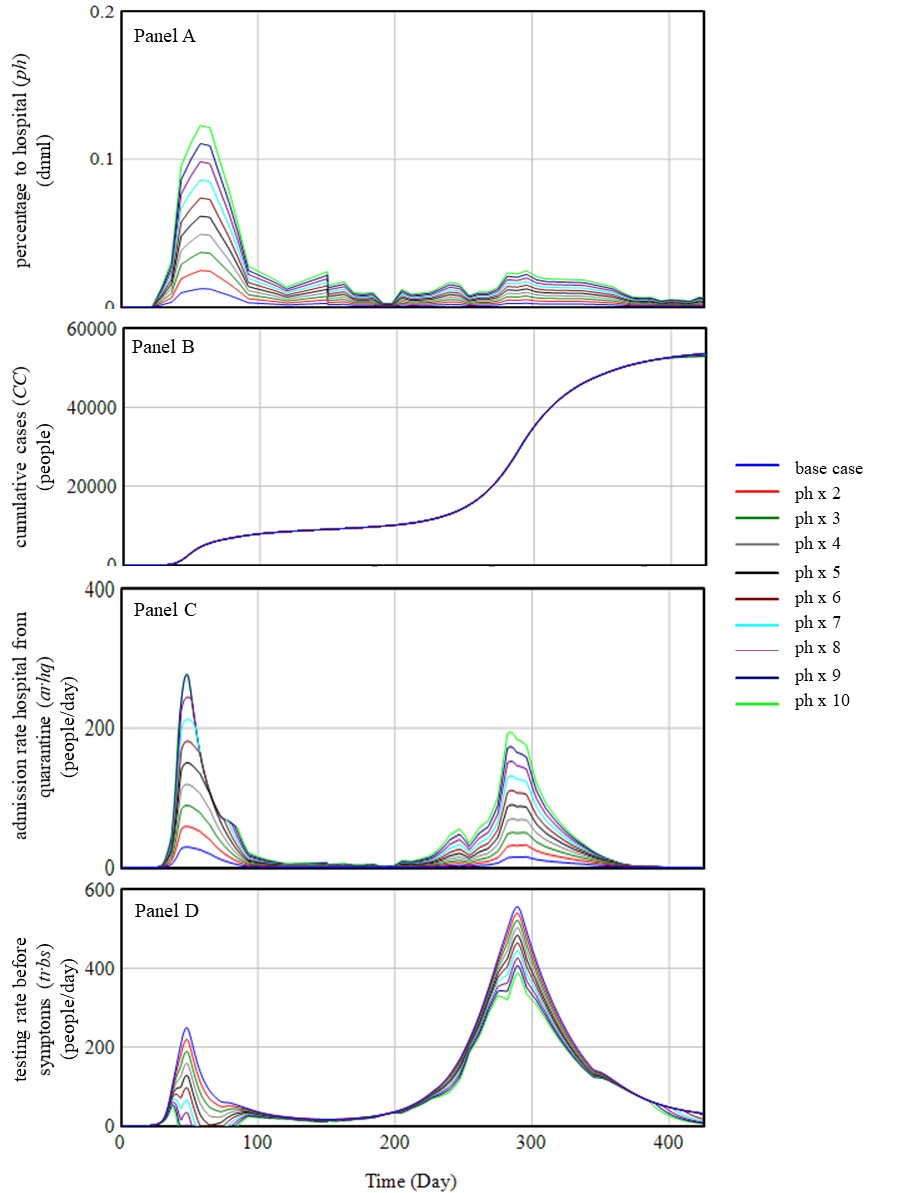
**

**Figure M. Robustness of model to extreme values of percentage to hospital (*ph*)**

**5.1.4 Introducing new variants (mutants) of COVID-19**

We have assumed that the normal infectivity (*i*) of COVID-19 remains constant during the 425 days of our simulations. Note that the effective infectivity (*effi*) changes over time though, as this variable depends on policy interventions (the hygiene focus factor, *H*). Here, we will check the robustness of our model to changes in the normal infectivity, like the introduction of a new variant or mutant. Assuming that such a mutant will increase the normal infectivity, we would expect the infection rate to increase as well, and as such mutants are not foreseen, we also expect to see higher inventory shortages. We simulate two situations: the introduction of a new variant on day 250 (when there is an inventory surplus), and on day 305 (when there is an inventory shortage). After testing the “base case” in these two situations, we also simulate what happens if Norway accepts/donates 100% of its diagnostic test inventory shortage/surplus (“best case”).

The introduction of the mutant is modeled as a first-order delay, such that the normal infectivity (i) gradually increases to a higher value. Furthermore, we assume that the new mutant has a 33% higher infectivity compared to the initial normal infectivity. Therefore,

$i\left( t \right)=0.455379+DELAY1(STEP\left( 0.3333*0.455379,250 \right),7)$ dmnl

When the mutant arrives on day 305, the number 250 in the equation above is replaced by 305.

The results are shown in Table K and Figure N.

**Table K. Comparison of scenarios with mutants at different times**

| **Scenario** | **Days in Lockdown Wave 2** | **Total COVID-19 Cases** | **Max. Infectious Population in Hospital Wave 1 or 2** | **Total deaths** | **Total Tests Shipped to Norway** |
| --- | --- | --- | --- | --- | --- |
| base case | 67.0 | 53740.1 | W1: 187.5 | 499.0 | 3366110 |
| base case mutant from day 250 | 95.1 | 80919.9 | W2: 215.8 | 700.4 | 5066570 |
| +100% of short. & surpl. mutant from day 250 | 100.2 | 79733.4 | W2: 191.6 | 573.6 | 4992510 |
| -100% of short. & surpl. mutant from day 250 | 90.0 | 77299.6 | W2: 205.5 | 680.7 | 4839930 |
| ±100% of short. & surpl. mutant from day 250 | 94.9 | 77437.1 | W2: 185.5 | 561.8 | 4848720 |
|  |  |  |  |  |  |
| base case mutant from day 305 | 116.2 | 67772.8 | W1: 187.5 | 540.5 | 4248130 |
| +100% of short. & surpl. mutant from day 305 | 127.4 | 64011.1 | W1: 113.3 | 409.7 | 4013630 |
| -100% of short. & surpl. mutant from day 305 | 109.4 | 65125.9 | W1: 187.7 | 539.3 | 4082270 |
| ±100% of short. & surpl. mutant from day 305 | 123.9 | 62798.2 | W1: 113.5 | 410.5 | 3937660 |

These results show that our model is capable of simulating mutants that increase the infectivity of COVID-19. We also find that the time of entry of the mutant has a high impact on infection rates. If the mutant arrives on day 305 the situation gets worse due to the increased infections, but the negative effects are not so devastating, as Norway had sufficient diagnostic tests at that time. If the mutant arrives on day 250, during an inventory shortage, it is a different story. This leads to an increase of hospitalizations in the second wave that is much higher than in first wave. In both situations, we also find that our best case (accepting and donating diagnostic tests) is still much better than the base case.

Note that Panel B in Figure N shows how the expectations or perceptions of policymakers about the contact rate times infectivity (*eci*) of the virus lags behind the real value (*ci*). First, *eci* is lower than ci when the mutant enters Norway. Later, *eci* is higher than *ci* as the impact of a new lockdown is not perceived yet.


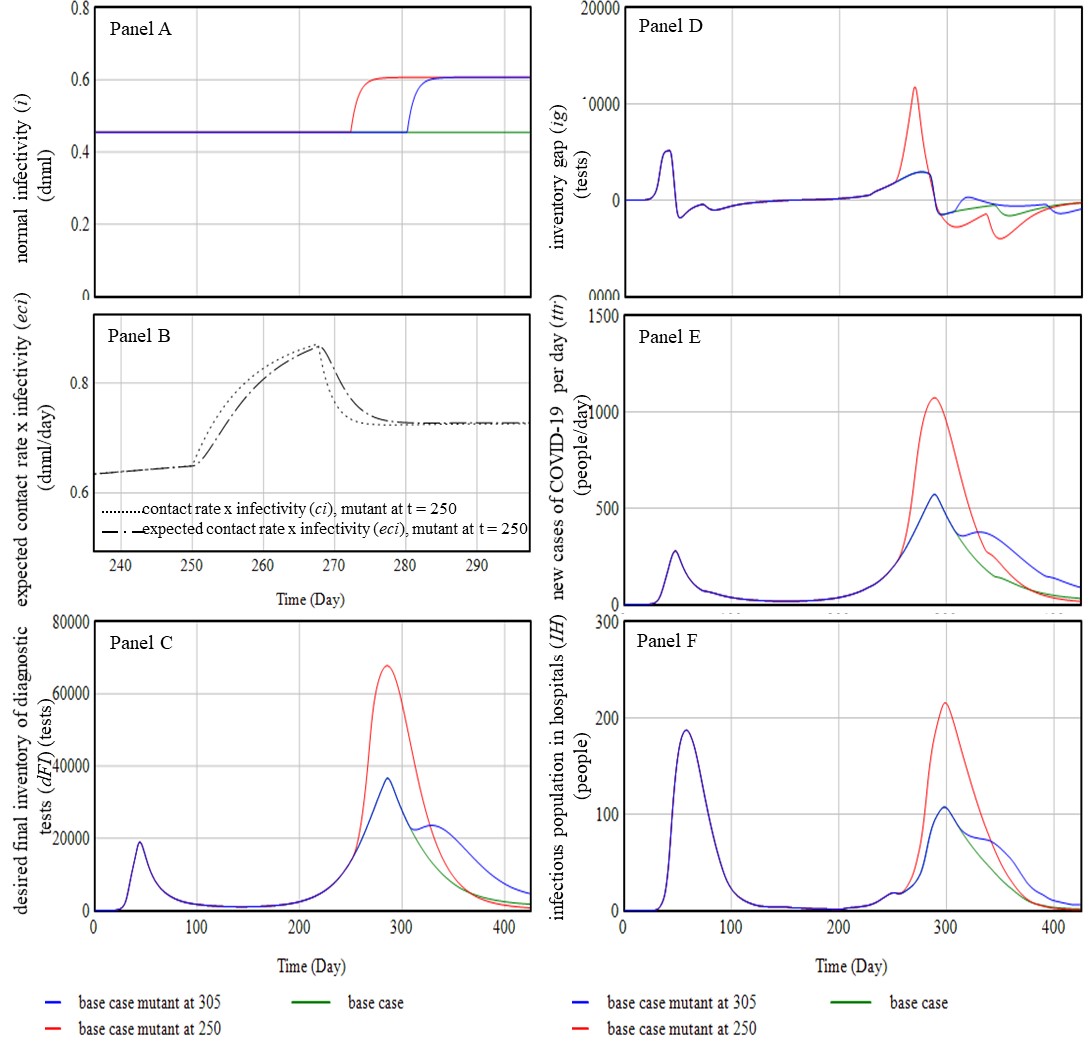


**Figure N. Introducing a mutant at two different times**

**5.2 Sensitivity analyses**

We have performed six sensitivity analyses with our model. The first three explore the impact of changes in typical supply chain parameters: the average shipment time of diagnostic tests (*ast*), the diagnostic test reallocation time (*trt*), and the benefits of having (and using) a prepositioned stock (*PFI*) of diagnostic tests. The fourth analysis explores the effects of different values of the percentage of the population that develops symptoms (*pds*). The fifth analysis looks at the impact of an earlier versus later lockdown in the first wave, by changing the threshold (*tsL1*). Finally, the sixth analysis explores the impact of increasing the number of diagnostic tests per person (*tpp*).

**5.2.1 Impact of the average shipment time of diagnostic tests (*ast*)**

We have assumed that the average shipment time of diagnostic tests from the factory to Norway is 3 days. Here, we will evaluate what the impact is of a shorter (1 day) and longer (5 days) average shipment time. Although we are not analyzing the costs and benefits of having local production of diagnostic tests in Norway, the simulation results with an average shipment time of 1 day can be a good indicator of the benefits of local production (assuming all other decision-making processes and delays with respect to diagnostic test production are the same, regardless of where you produce these diagnostic tests). For each of the three different values of the average shipment time, we have simulated the base case and our best case (accepting/donating 100% of inventory shortage/surplus). The results are presented in Table L and Figure O. These results show that shorter average shipment times are much better than longer average shipment times, which is not really a surprising result as longer delays in receiving diagnostic tests only fuels the exponential growth of the infection rate. This shows the benefits of (more) local production. However, it is surprising to see that the collaborative scenario (accepting/donating 100% of shortage/surplus) with a long average shipment time (5 days) is better that the scenario without collaboration and a short average shipment time (1 day). These results suggest that local production is good, but collaboration is better. Our analyses also suggest that even with different values of the average shipment time, our general recommendation stands; the collaborative, or best case scenario remains the best case scenario.

**Table L. Comparison of in scenarios with different average shipment times**

| **Scenario** | **Days in Lockdown Wave 2** | **Total COVID-19 Cases** | **Max. Infectious Population in Hospital Wave 1** | **Total deaths** | **Total Tests Shipped to Norway** |
| --- | --- | --- | --- | --- | --- |
| base case ast1 | 30.8 | 52436.5 | 147.6 | 442.7 | 3284390 |
| base case ast3 | 38.0 | 53740.1 | 187.5 | 499.0 | 3366110 |
| base case ast5 | 39.3 | 54849.1 | 254.4 | 517.1 | 3436300 |
| ±100% of short. & surpl. ast1 | 23.8 | 51248.5 | 101.5 | 362.7 | 3210140 |
| ±100% of short. & surpl. ast3 | 25.2 | 51692.2 | 113.5 | 385.3 | 3237880 |
| ±100% of short. & surpl. ast5 | 26.9 | 51940.6 | 127.5 | 412.6 | 3253610 |

**
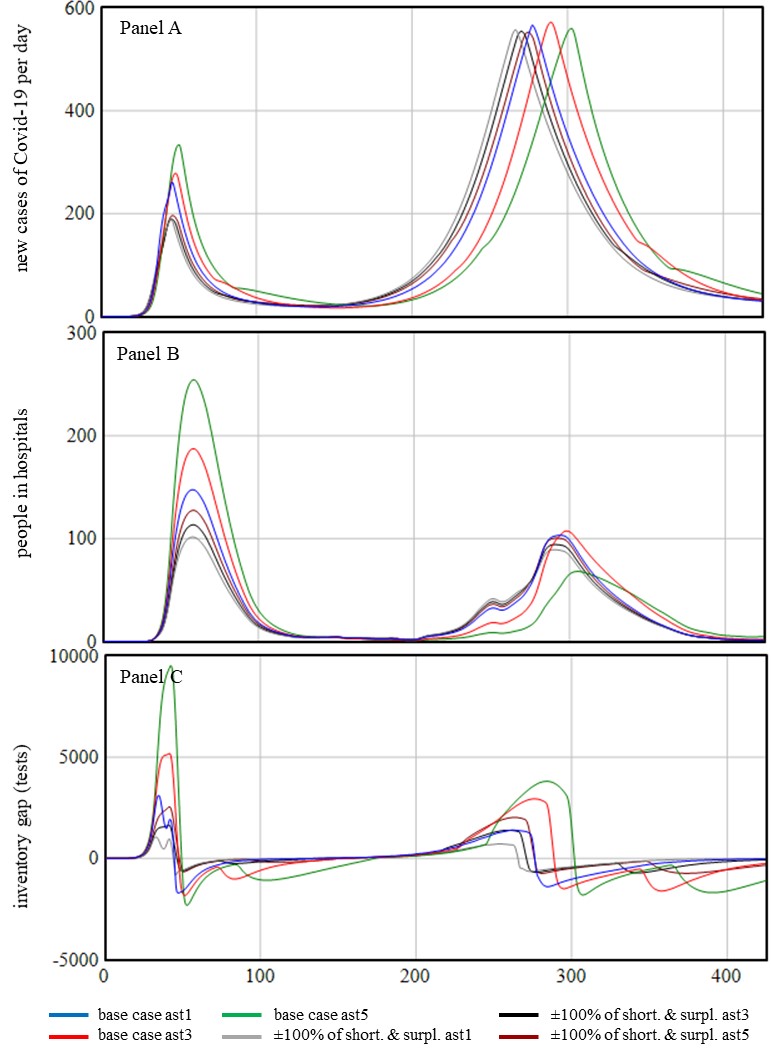
**

**Figure O. Comparison of scenarios with different average shipment times**

**5.2.2 Impact of the diagnostic test reallocation time (*trt*)**

In our model we have assumed that when countries offer to give diagnostic tests to Norway, that the average time it takes to reallocate these diagnostic tests from that country to Norway is only 1 day. We also assume that it takes the same time to donate diagnostic tests from Norway to another country. As this is already a very short time, we believe it makes no sense analyzing the effects of even shorter test reallocation times. Yet it is interesting to find out if the test reallocation times can become so long that collaborating with other countries is no longer beneficial. We have therefore analyzed scenarios with test reallocation times ranging from 1 day (base case) to 20 days and we compare these scenarios with our base case without collaboration. The results are presented in Table M and Figure P. It is not surprising that for longer test reallocation times, the benefits of collaboration are reduced. With a *trt* of 20 days, we get 53491 COVID-19 cases, compared to 51692 cases with a *trt* of 1 day (an increase of 3.5%). And with a *trt* of 20 days, we get 490 deaths, compared to 385 deaths with a *trt* of 1 day (an increase of 27.2%). Nevertheless, even when collaboration takes as long as 20 days, it is still slightly better than no collaboration (base case). This suggest that accepting and donating diagnostic tests is the preferred scenario, even if it takes a long time to operationalize such collaboration.

**Table M. Comparison of scenarios with different diagnostic test reallocation times**

| **Scenario** | **Days in Lockdown Wave 2** | **Total COVID-19 Cases** | **Max. Infectious Population in Hospital Wave 1** | **Total deaths** | **Total Tests Shipped to Norway** |
| --- | --- | --- | --- | --- | --- |
| base case | 38.0 | 53740.1 | 187.5 | 499.0 | 3366110 |
| ±100% of short. & surpl. trt1 | 25.2 | 51692.2 | 113.5 | 385.3 | 3237880 |
| ±100% of short. & surpl. trt2 | 28.0 | 52239.3 | 133.5 | 419.8 | 3272090 |
| ±100% of short. & surpl. trt3 | 29.6 | 52549.9 | 145.1 | 438.8 | 3291520 |
| ±100% of short. & surpl. trt4 | 30.4 | 52746.8 | 152.4 | 450.4 | 3303840 |
| ±100% of short. & surpl. trt8 | 32.3 | 53122.1 | 167.7 | 472.9 | 3327330 |
| ±100% of short. & surpl. trt10 | 32.8 | 53203.2 | 170.5 | 477.0 | 3332410 |
| ±100% of short. & surpl. trt14 | 34.1 | 53361.4 | 175.9 | 484.8 | 3342330 |
| ±100% of short. & surpl. trt20 | 35.6 | 53491.2 | 180.0 | 490.3 | 3350480 |

**
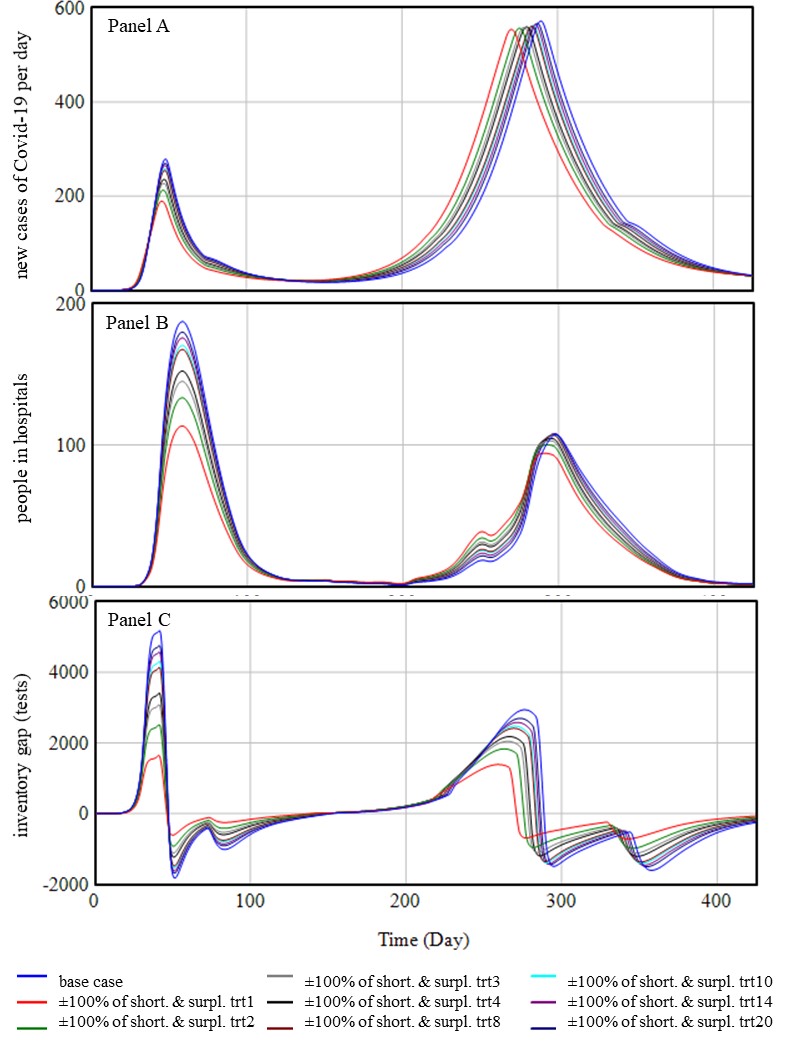
**

**Figure P. Comparison of scenarios with different diagnostic test reallocation times**

**5.2.3 Impact of having and using a predispositioned stock of diagnostic tests (*PFI*)**

Having a prepositioned stock of diagnostic tests is an addition to our model, instead of changing the value of a parameter in our model. But we could say, of course, that the value is 0 in our base case and that we experiment with another value in this analysis. To model a predispositioned (or safety) stock of diagnostic tests, we had to add another stock to our model, this is shown in Figure P. The prepositioned stock (PFI) is in a way an extra stock of diagnostic tests Norway has access to, from the start of the pandemic until the stock is empty. Son instead of accepting tests from a different country when there is a diagnostic test shortage, Norway can use (parts) of its own safety stock (PFI) when there is a shortage.

**
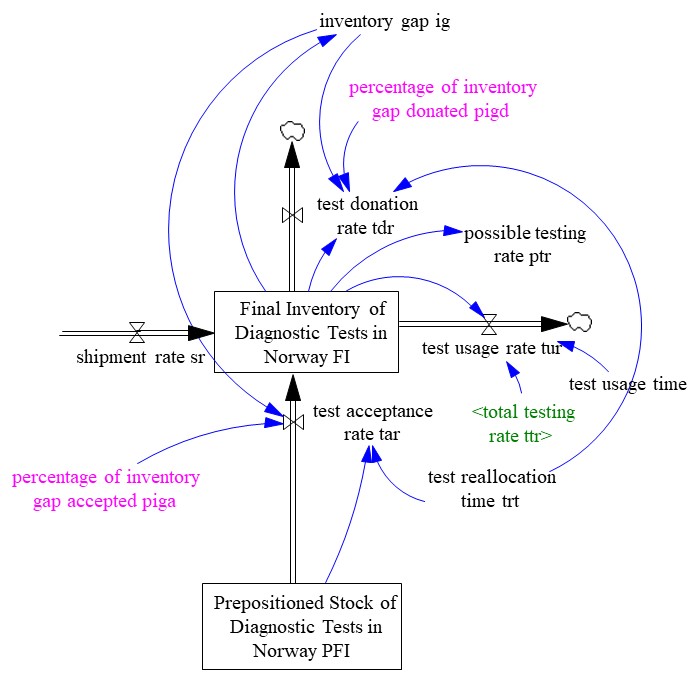
**

**Figure Q. Extension of the model to include prepositioning of diagnostic tests**

The additional equations to model *PFI* are given below:

$PFI\left( t \right)=PFI\left( 0 \right)+\int_{0}^{t} -tar\left( s \right)ds;PFI\left( 0 \right)=50000$ tests

$tar\left( t \right)=MIN(PFI(t)/trt,piga*MAX(0,ig\left( t \right))/trt)$ dmnl/day

$trt=0.25$ days

*PFI* is modeled as a stock and is defined by the integral of its outflow: the test acceptance rate (*tar*). Initially, we assume an inventory level of 50000 diagnostic tests. This is sufficient to cover the inventory gaps during the first wave, but only partially sufficient to cover the inventory gaps in the second wave. The equation of *tar* has not been changed with respect to our base case. But we have shortened the *trt* to only 0.25 days as this *PFI* is assumed to be ready for use in Norway which could shorten average reallocation times dramatically. We also assume that *PFI* is not replenished after use. So, it is like an emergency stock, you can use the stock in case of a shortage, but otherwise, the normal order and delivery procedures apply. The scenarios that we have simulated in this analysis is the way the *PFI* is used. Policymakers could decide to use as much as they need, all the time, until the stock is empty, or they could be more cautious during the first wave to save some diagnostic tests for the second wave. This decision is operationalized by our variable percentage of inventory gap accepted (*piga*). When *piga* is 1 or 100%, every time an inventory gap is discovered, the entire size of this gap is taken out of *PFI*. When *piga* is 0.5, only 50% of the inventory gap is replenished and taken out of the *PFI*.

The results are presented in Table N and Figure R for different values of *piga*, or the % of the inventory gap that is covered by *PFI*. We compare having and using a *PFI* with our original base case and our best case (accepting/donating 100% of shortage/surplus). The results indicate that when policymakers are too careful with using the *PFI* (when they only replenish 7% of the inventory gap, and, in doing so, use this “emergence stock” very carefully), they will end up with a surplus of diagnostic tests after the second wave is over. Using as much as possible whenever it is needed (replenishing max 100% of the inventory gap from the *PFI*) outperforms the other scenarios. It leads to large benefits in the first wave, and it does not make the second wave worse than it was in the base case. The scenario in which only 50% of the inventory gap is replenished by the *PFI* performs similar to our best case (accepting/donating 100% of shortage/surplus). The max 100% of PFI scenario is even better than this best case. This is due to the very short test reallocation times that apply when Norway has its own prepositioned stock of diagnostic tests. Overall, our results suggest that prepositioning helps. But this implies of course that it is known what kind of diagnostic tests you need for the next pandemic. Without knowing this, our collaborative best case scenario seems to outperform prepositioning.

**Table N. Comparison of scenarios with different distributions of prepositioned stock**

| **Scenario** | **Days in Lockdown Wave 1** | **Total COVID-19 Cases** | **Max. Infectious Population in Hospital Wave 1** | **Total deaths** | **Total Tests Shipped to Norway (incl. 50000)** | **Tests remaining in prepositioned stock** |
| --- | --- | --- | --- | --- | --- | --- |
| base case | 38.0 | 53740.1 | 187.5 | 499.0 | 3366110 | 0.0 |
| ±100% of short. & surpl. | 25.2 | 51692.2 | 113.5 | 385.3 | 3237880 | 0.0 |
| max 5% of PFI | 31.4 | 53297.2 | 157.4 | 454.5 | 3388300 | 18548.3 |
| max 7% of PFI | 30.4 | 53207.5 | 148.8 | 440.7 | 3382690 | 9269.0 |
| max 10% of PFI | 29.6 | 53120.3 | 140.2 | 426.3 | 3377250 | 0.0 |
| max 50% of PFI | 24.2 | 52760.6 | 97.9 | 356.6 | 3348970 | 0.0 |
| max 100% of PFI | 22.6 | 52492.0 | 87.2 | 332.5 | 3337780 | 0.0 |

**
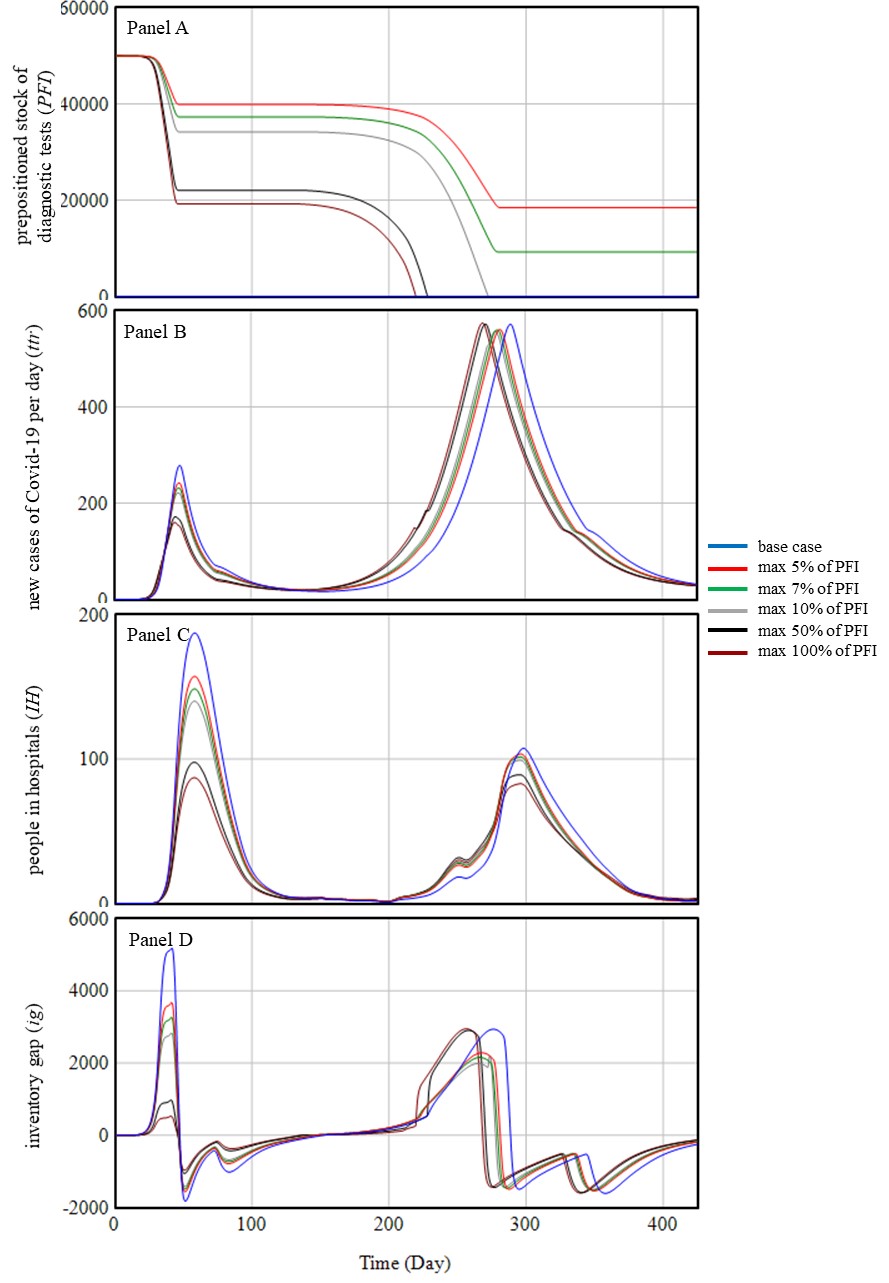
**

**Figure R. Comparison of scenarios with different distributions of prepositioned stock**

**5.2.4 Impact of the percentage of the population that develops symptoms (*pds*)**

The percentage of the population that develops symptoms of COVID-19 (pds) divides the susceptible population into the infectious pre-symptomatic population and the infectious asymptomatic population. We have assumed that the asymptomatic population has the same likelihood (contact rate and infectivity) of infecting the susceptible population as the pre-symptomatic population. The “benefits” of more asymptomatic people is that these people do not need to be admitted to hospitals and as they never get any symptoms, we assume that they will not get tested. This has the side-effect that it is unclear how many people are infected which could lead policymakers to underestimate the severity of the pandemic. We have tested four additional scenarios with four different values of *pds*: 30%, 35%, 40% (the value used in the base case), 45% and 50%.

The results are presented in Table O and Figure S. The results are intuitive: the higher the percentage of the population that develops symptoms, the higher the total number of COVID-19 cases, the higher the maximum occupation in hospitals, the higher the number of deaths and the higher the number of diagnostic tests used. Looking at the behavior over time (Figure S) we see that it takes a longer time for the second wave to hit the country when the *pds* is lower. This is because for lower values of *pds* the number of hidden (asymptomatic) infections is higher, which causes a delay in discovering the impact of the pandemic.

**Table O. Comparison of scenarios with different percentages that develops symptoms (*pds*)**

| **Scenario** | **Days in Lockdown Wave 1** | **Total COVID-19 Cases** | **Max. Infectious Population in Hospital Wave 1** | **Total deaths** | **Total Tests Shipped to Norway** |
| --- | --- | --- | --- | --- | --- |
| base case with pds 30% | 37.9 | 45680.8 | 175.2 | 429.8 | 2860550 |
| base case with pds 35% | 38.6 | 49263.4 | 182.4 | 461.5 | 3085360 |
| base case with pds 40% | 38.0 | 53740.1 | 187.5 | 499.0 | 3366110 |
| base case with pds 45% | 37.3 | 58414.0 | 196.3 | 534.5 | 3659330 |
| base case with pds 50% | 36.1 | 62544.9 | 205.8 | 565.7 | 3918270 |


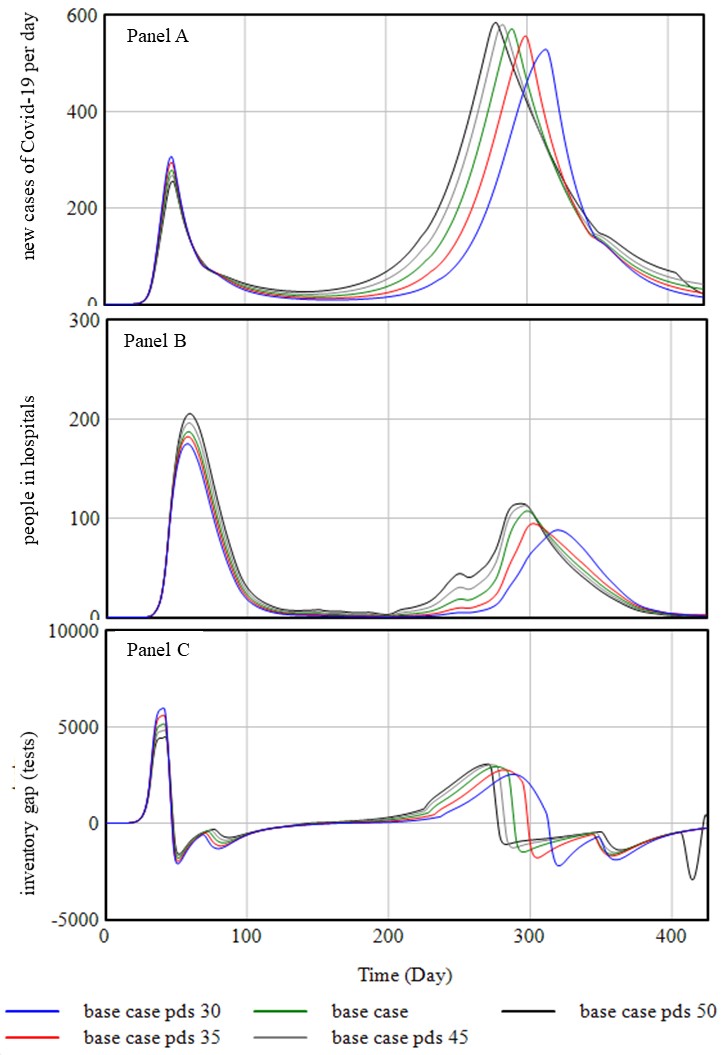


**Figure S. Comparison of scenarios with different percentages that develops symptoms (*pds*)**

**5.2.5 Impact of an earlier versus later lockdown in the first wave (*tsL1*)**

Both policy interventions and diagnostic tests are ways to flatten the curve. Norway was relatively early in locking down society on March 12, 2020. At that time, the total number of COVID-19 cases (*CC*) was almost 900 people. We have modeled the start of the lockdown in this first wave by using a threshold (*tsL1*). This threshold has the value of 895 people in the base case, leading to a lockdown on day 41 (as day 1 is February 1, 2020, day 41 is March 12, 2020). To simulate the effects of an earlier and later start of lockdown 1, we have simulated two additional scenarios: one with a lower (145 people) and one with a higher (2571 people) threshold. The former leads to a lockdown that starts a week earlier (day 34) than in the base case, the latter leads to a lockdown that starts a week later (day 48) than in the base case. We have not changed the thresholds for ending lockdown 1. Also, the thresholds for lockdown 2 are kept the same as in the base case.

The results are presented in Table P and Figure T. These results confirm the basic behavior of the reinforcing contagion loop. When a reinforcing loop starts to spiral out of control, the sooner the growth is dampened or stopped the better. Locking down society one week earlier seems to have enormous beneficial effects for all subsystems of our model: shorter lockdowns, less infected people, less pressure in hospitals, fewer deaths, and fewer diagnostic tests required. In Table P we also included our best case (accepting/donating 100% of shortage/surplus). Locking down society one week earlier outperforms this best case. This suggests that when countries cannot collaborate with others, or have problems getting access to diagnostic tests, they should be very early in implementing policy interventions, as good and timely interventions can compensate for a lack of diagnostic tests.

**Table P. Comparison of scenarios with different thresholds for start of Lockdown 1 (*tsL1*)**

| **Scenario** | **Days in Lockdown Wave 1** | **Total COVID-19 Cases** | **Max. Infectious Population in Hospital Wave 1** | **Total deaths** | **Total Tests Shipped to Norway** |
| --- | --- | --- | --- | --- | --- |
| base case early start of L1 | 22.9 | 51288.0 | 87.9 | 335.6 | 3212340 |
| base case | 38.0 | 53740.1 | 187.5 | 499.0 | 3366110 |
| base case late start of L1 | 51.6 | 56682.3 | 361.0 | 578.9 | 3551340 |
| ±100% of short. & surpl. | 25.2 | 51692.2 | 113.5 | 385.3 | 3237880 |

**
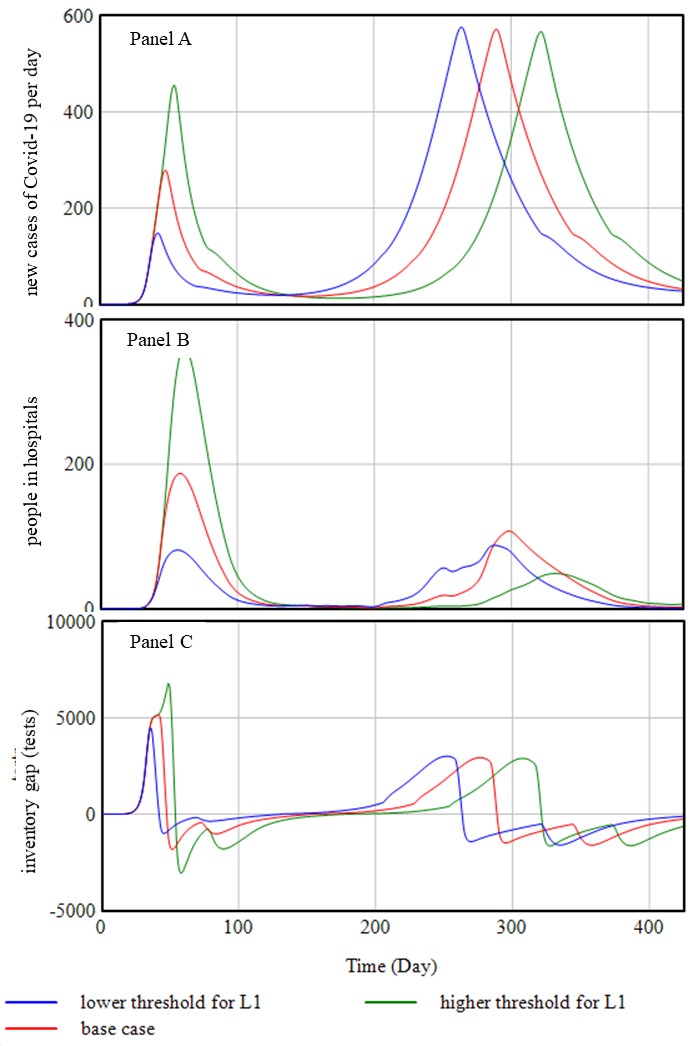
**

**Figure T. Comparison of scenarios with different thresholds for start of Lockdown 1 (*tsL1*)**

**5.2.6 Impact of the number of diagnostic tests per person (*tpp*)**

In our model, we have assumed that after a person is tested positively, this person will not be tested again after a few weeks to check if that person is still infectious or not. If each person would require two or more tests, we would need to change the variable diagnostic tests per person (*tpp*). We have simulated the effects of a higher *tpp* (2, 3, and 4 tests per person) compared to our base case (1 test per person). And we have simulated our best case (accepting/donating 100% of shortage/surplus) also with a *tpp* of 4.

The results are presented in Table Q and Figure U. The results are straightforward. When more tests per person are required, more tests per person are ordered, so the shipment rates to Norway are increased (as we assume that the factory has infinite access to material needed to produce diagnostic tests). Higher values of *tpp* lead to higher inventory levels, and higher inventory gaps, but this does not impact the behavior of the COVID-19 transmission subsystem and the policy intervention subsystem. The best case with a *tpp* of 4 also leads to the same results as the best case with a *tpp* of 1.

**Table Q. Comparison of scenarios with different number of tests per person (*tpp*)**

| **Scenario** | **Days in Lockdown Wave 1** | **Total COVID-19 Cases** | **Max. Infectious Population in Hospital Wave 1** | **Total deaths** | **Total Tests Shipped to Norway** |
| --- | --- | --- | --- | --- | --- |
| base case | 38.0 | 53740.1 | 187.5 | 499.0 | 3366110 |
| base case with tpp2 | 38.0 | 53740.1 | 187.5 | 499.0 | 6732220 |
| base case with tpp3 | 38.0 | 53740.1 | 187.5 | 499.0 | 10098300 |
| base case with tpp4 | 38.0 | 53740.1 | 187.5 | 499.0 | 13464400 |
| ±100% of short. & surpl. | 25.2 | 51692.2 | 113.5 | 385.3 | 3237880 |
| ±100% of short. & surpl. & tpp4 | 25.2 | 51692.2 | 113.5 | 385.3 | 12951500 |

**
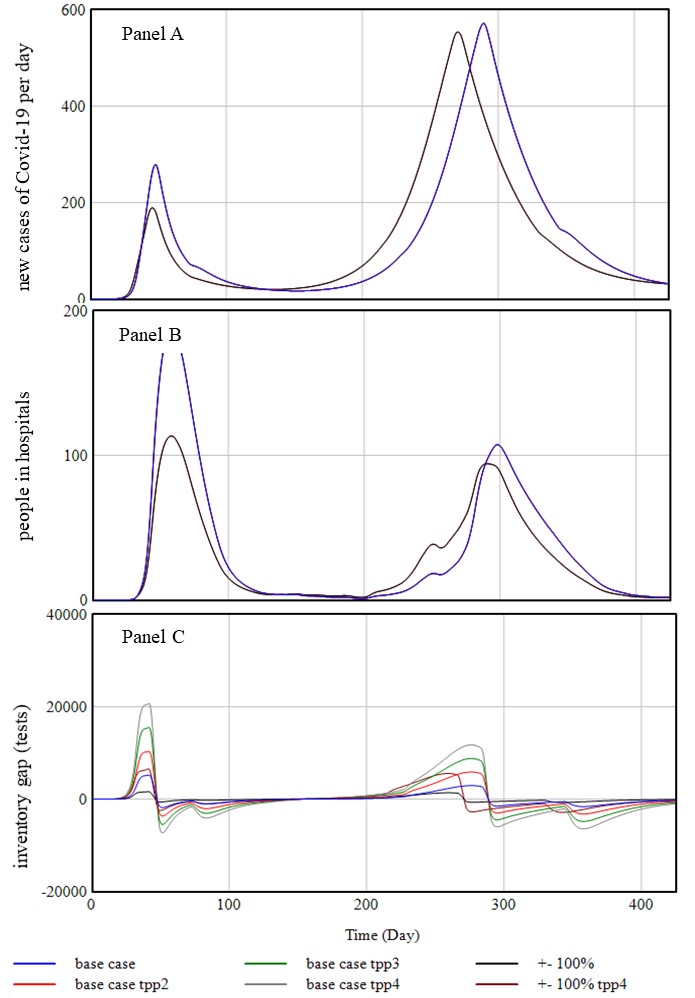
**

**Figure U. Comparison of scenarios with different number of tests per person (*tpp*)**

**Appendix 6. Model Assessment Results**

Table R presents the model assessment results (Martinez-Moyano, 2012). The tool provides enhanced transparency and assessment through a simple upload and report generation of our Vensim model. This tool allowed us to determine warnings and potential omissions. We reviewed these concerns identified by the tool – and deemed them acceptable due to purpose of our model. Note that the largest number of warnings are because of “undocumented equations”. This was a conscious decision, as we decided to document our equations in Appendix 1-3 of this e-companion, instead of hidden in the Vensim model. As such, our equations can be viewed by anyone and not just readers with access to the Vensim software.

**Table R. Model assessment results of the permanent beta simulation model**

| **Model Information** | **Number** |
| --- | --- |
| Total Number of Variables | 213 |
| Total Number of State Variables (Level+Smooth+Delay Variables) | 39 (18.3%) |
| Total Number of Stocks (Stocks in Level+Smooth+Delay Variables) | 35 (16.4%) |
| Total Number of Macros | 0 |
| Variables with Source Information | 0 |
| Variables with Dimensionless Units | 76 (35.7%) |
| Variables without Predefined Min or Max Values | 211 (99.1%) |
| Function Sensitivity Parameters | 0 |
| Data Lookup Tables | 0 |
| Time Unit | Day |
| Initial Time | 1 |
| Final Time | 425 |
| Reported Time Interval | TIME STEP |
| Time Step | 0.0625 |
| Model Is Fully Formulated | Yes |
| Modeler-Defined Groups | - No - |
| VPM File Available | - No - |

| **Warnings** | **Number** |
| --- | --- |
| Undocumented Equations | 213 (100%) |
| Equations with Embedded Data | 0 |
| Equations With Unit Errors or Warnings | Unavailable |
| Variables Not in Any View | 0 |
| Incompletely Defined Subscripted Variables | 0 |
| Nonmonotonic Lookup Functions | 1 (0.5%) |
| Cascading (Chained) Lookup Functions | 0 |
| Non-Zero End Sloped Lookup Functions | 1 (0.5%) |
| Equations with "IF THEN ELSE" Functions | 20 (9.4%) |
| Equations with "MIN" or "MAX" Functions | 13 (6.1%) |
| Equations with "STEP", "PULSE", or Related Functions | 0 |

| **Potential Omissions** | **Number** |
| --- | --- |
| Unused Variables | 14 |
| Supplementary Variables | 0 |
| Supplementary Variables Being Used | 0 |
| Complex Variable Formulations (Richardson's Rule = 3) | 26 |
| Complex Stock Formulations | 0 |

**Appendix 7. Listing All Variables with Values and Units**

| **Symbol** | **Description** | **Value** | **Units** |
| --- | --- | --- | --- |
| ahtic | average hospital time before ICU | 4 | days |
| ahtr | average hospital time before recovery | 8 | days |
| aict | average ICU time | 12 | days |
| aift | average infectivity time | 5 | days |
| air | asymptomatic infection rate |  | people/day |
| ait | average isolation time | 14 | days |
| aith | average isolation time before hospitalization | 8 | days |
| apt | average production time | 3 | days |
| aqt | average quarantine time | 14 | days |
| aqth | average quarantine time before hospitalization | 6 | days |
| arhi | admission rate hospital from isolation |  | people/day |
| arhq | admission rate hospital from quarantine |  | people/day |
| aric | admission rate intensive care |  | people/day |
| ast | average shipment time | 3 | days |
| atR | average time used for R calculation | 2.25 | days |
| att | average test time | 1 | days |
| bH1 | period before H1 |  | dmnl |
| bH1H2 | period between H1 and H2 |  | dmnl |
| bH2H3 | period between H2 and H3 |  | dmnl |
| bL1 | period before L1 |  | dmnl |
| bL1L2 | period between L1 and L2 |  | dmnl |
| bL2L3 | period between L2 and L3 |  | dmnl |
| c | normal contact rate | 3.09763 (*) | people/people/day |
| CC | cumulative cases |  | people |
| cDEL1 | change end day of lockdown 1 |  | dmnl/day |
| cDEL2 | change end day of lockdown 2 |  | dmnl/day |
| cDSH1 | change start day hygiene focus 1 |  | dmnl/day |
| cDSH2 | change start day hygiene focus 2 |  | dmnl/day |
| cDSL1 | change start day lockdown 1 |  | dmnl/day |
| cDSL2 | change start day lockdown 2 |  | dmnl/day |
| cFDTR | change of forecast of diagnostic test rate |  | people/day/day |
| cH | change hygiene focus factor |  | dmnl/day |
| ci | contact rate x infectivity |  | dmnl/day |
| cL | change lockdown factor |  | dmnl/day |
| cPci | change in Perceived ci |  | dmnl/day/day |
| cPF | change of production forecasts |  | tests/day/day |
| cPTci | change in Perceived Trend ci |  | dmnl/day |
| cRci | change in Reference ci |  | dmnl/day/day |
| dchf | delay in changing hygiene focus | 598.431 (*) | days |
| DEL1 | day to end lockdown 1 |  | dmnl |
| DEL2 | day to end lockdown 2 |  | dmnl |
| dFI | desired final inventory |  | tests |
| dFIF | desired final inventory factory |  | tests |
| dH1 | period during H1 |  | dmnl |
| dH2 | period during H2 |  | dmnl |
| dL1 | period during L1 |  | dmnl |
| dL2 | period during L2 |  | dmnl |
| dou | delay in opening up | 158.778 (*) | days |
| DP | deceased population |  | people |
| dpr | desired production rate |  | tests/day |
| dprRM | desired purchasing rate raw material |  | tests/day |
| dr | death rate |  | people/day |
| dRM | desired raw material inventory |  | tests |
| drs | discovery rate of symptoms |  | people/day |
| DSH1 | day to start hygiene focus 1 |  | dmnl |
| DSH2 | day to start hygiene focus 2 |  | dmnl |
| DSL1 | day to start lockdown 1 |  | dmnl |
| DSL2 | day to start lockdown 2 |  | dmnl |
| dsr | desired shipment rate |  | tests/day |
| dtms | delay in taking measures seriously | 2 | days |
| dtrbs | desired testing rate before symptoms |  | people/day |
| e1 | end of wave 1 | 151 | days |
| e2 | end of wave 2 | 350 | days |
| eair | expected asymptomatic infection rate |  | people/day |
| eci | expected ci |  | dmnl/day |
| edrs | expected discovery rate of symptoms |  | people/day |
| eeL1 | earliest possible end day of lockdown 1 |  | dmnl |
| eeL2 | earliest possible end day of lockdown 2 |  | dmnl |
| effc | effective contact rate |  | dmnl/day |
| effi | effective infectivity |  | dmnl |
| eH1 | end day of increased hygiene focus 1 |  | dmnl |
| eH2 | end day of increased hygiene focus 2 |  | dmnl |
| EIAP | expected infectious asymptomatic population |  | people |
| eiIPP | expected initial IPP | 0.292285 (*) | people |
| EIPP | expected infectious pre-symptomatic population |  | people |
| EIQ | expected infectious population in quarantine |  | people |
| eL1 | end day of lockdown 1 |  | dmnl |
| eL2 | end day of lockdown 2 |  | dmnl |
| epir | expected pre-symptomatic infection rate |  | people/day |
| ER | expected recovered population |  | people |
| eriIQ | expected relative infectivity IQ | 0.39917 (*) | dmnl |
| erra | expected recovery rate asymptomatic population |  | people/day |
| errq | expected recovery rate after quarantine |  | people/day |
| ES | expected susceptible population |  | people |
| exL1 | extent of lockdown 1 | 0.412605 (*) | dmnl |
| exL2 | extent of lockdown 2 |  | dmnl |
| fat | forecast adjustment time | 5.38964 (*) | days |
| FDTR | forecast of diagnostic test rate |  | test/day |
| ffat | factory forecast adjustment time | 1 | days |
| FI | final inventory of diagnostic tests in Norway |  | tests |
| fiat | factory inventory adjustment time | 1 | days |
| FIF | final inventory of diagnostic tests in factory |  | tests |
| gfdtr | gross forecast of diagnostic test rate |  | tests/day |
| H | hygiene focus factor |  | dmnl |
| hRci | time horizon Rci | 28 | days |
| i | normal infectivity i | 0.455379 (*) | dmnl |
| IAP | infectious asymptomatic population |  | people |
| iat | inventory adjustment time | 1 | days |
| ic | inventory coverage |  | tests |
| ig | inventory gap |  | tests |
| IH | infectious population in hospital |  | people |
| iH | initial hygiene focus | 1 | dmnl |
| iH1 | infectivity due to hygiene focus 1 | 0.51788 (*) | dmnl |
| II | infectious population in isolation |  | people |
| IIC | infectious population in intensive care |  | people |
| iL | initial lockdown factor | 1 | dmnl |
| initial IPP | initial infectious pre-symptomatic population | 0.313049 (*) | people |
| initial N | initial total population | 4947880 (*) | people |
| initial S | initial susceptible population | 2009110 (*) | people |
| iPci | initial perceived ci |  | dmnl/day |
| IPP | infectious pre-symptomatic population |  | people |
| IQ | infectious population in quarantine |  | people |
| itci | indicated trend ci |  | dmnl |
| L | lockdown factor |  | dmnl |
| lH1 | length of hygiene focus wave 1 | 91 | days |
| lH2 | length of hygiene focus wave 2 | 182 | days |
| lph | lookup percentage hospital |  | dmnl |
| mic | minimum inventory coverage | 0.157191 (*) | days |
| mlL1 | minimum length of lockdown wave 1 | 14 | days |
| mlL2 | minimum length of lockdown wave 2 | 42 | days |
| mph1 | multiplier percentage to hospital 1 | 0.151231 (*) | dmnl |
| mph2 | multiplier percentage to hospital 2 | 0.0906175 (*) | dmnl |
| mpic1 | multiplier percentage to ICU 1 | 2.68311 (*) | dmnl |
| mpic2 | multiplier percentage to ICU 2 | 8.08707 (*) | dmnl |
| N | total population |  | people |
| ndp | normal death percentage | 0.849583 (*) | dmnl |
| OB | order backlog |  | tests |
| ocr | order completion rate |  | tests/day |
| opt | order processing time | 1 | days |
| or | order rate |  | tests/day |
| Pci | perceived ci |  | dmnl/day |
| pds | percentage that develops symptoms | 0.4 | dmnl |
| PF | production forecast |  | tests/day |
| ph | percentage to hospital |  | dmnl |
| pic | percentage to IC |  | dmnl |
| piga | percentage of inventory gap accepted | scenario | dmnl |
| pigd | percentage of inventory gap donated | scenario | dmnl |
| pir | pre-symptomatic infection rate |  | people/day |
| pr | production rate pr |  | tests/day |
| PTci | perceived trend ci |  | dmnl |
| ptr | possible testing rate |  | people/day |
| ptrbs | possible testing rate before symptoms |  | people/day |
| pur | purchasing rate |  | tests/day |
| R | recovered population |  | people |
| Rci | Reference ci |  | dmnl/day |
| real CC | real data on cumulative cases |  | people |
| real DP | real data on deceased population |  | people |
| real IH | real data on infectious population in hospital |  | people |
| real IIC | real data on infectious population in IC |  | people |
| real R | real data on reproduction rate R |  | dmnl |
| real ttr | real data on total test rate |  | people/day |
| reL1 | real end of lockdown 1 | 79 | days |
| reL21 | real end of lockdown 1 | 350 | days |
| riIQ | relative infectivity of IQ | 0.473248 (*) | dmnl |
| RMF | raw material for diagnostic tests in factory |  | tests |
| rra | recovery rate asymptomatic population |  | people/day |
| rrh | recovery rate after hospital |  | people/day |
| rri | recovery rate after isolation |  | people/day |
| rric | recovery rate intensive care |  | people/day |
| rrq | recovery rate after quarantine |  | people/day |
| rsH1 | real start of hygiene focus 1 | 31 | days |
| rsH2 | real start of hygiene focus 2 | 198 | days |
| rsL1 | real start of lockdown 1 | 41 | days |
| rsL2 | real start of lockdown 2 | 283 | days |
| S | susceptible population |  | people |
| scp | signal to change policy | 1 | dmnl/day |
| sevL2 | severity of lockdown 2 | 1.62589 (*) | dmnl |
| sH1 | start day of increased hygiene focus 1 |  | dmnl |
| sH2 | start day of increased hygiene focus 2 |  | dmnl |
| sL1 | start day of lockdown 1 |  | dmnl |
| sL2 | start day of lockdown 2 |  | dmnl |
| sr | shipment rate |  | tests/day |
| tar | test acceptance rate |  | tests/day |
| tdr | test donation rate |  | tests/day |
| teL1 | threshold for end of lockdown 1 | 66.04 | people/day |
| teL2 | threshold for end of lockdown 2 | 134.55 | people/day |
| titci | time for indicated trend ci | 28 | dmnl |
| tPci | time to perceive ci | 2 | days |
| tpp | tests per person | 1 | tests/people |
| tPTci | time to perceive trend in ci | 28 | days |
| trbs | testing rate before symptoms |  | people/day |
| trt | test reallocation time | 1 | days |
| tsH1 | threshold for start of hygiene focus 1 | 55.51 | people |
| tsH2 | threshold for start of hygiene focus 1 | 34.22 | people/day |
| tsL1 | threshold for start of lockdown 1 | 896.40 | people |
| tsL2 | threshold for start of lockdown 1 | 524.35 | people/day |
| ttp | target test percentage | 0.0159745 | dmnl |
| ttr | total testing rate |  | tests/day |
| tur | test usage rate |  | tests/day |
| tut | test usage time | 1 | day |

(*) means the value was found through calibration

**Appendix 8. References**

Martinez-Moyano, I.J. 2012. Documentation for model transparency. *System Dynamics Review* **28**(2) 199–208.

Oliva, R. 2003. Model calibration as a testing strategy for system dynamics models. *European Journal of Operational Research* **151**(3) 552-568.

Sterman, J. D. 1984. Appropriate summary statistics for evaluating the historical fit of system dynamics models. *Dynamica* **10**(2) 51–66.

Sterman, J. D. 2000. *Business Dynamics: Systems Thinking and Modeling for a Complex World.* Irwin/McGraw-Hill, Boston.
